# Modeling of Biological Activity of PEO-Coated Titanium Implants with Conjugates of Cyclic RGD Peptide with Amino Acid Bisphosphonates

**DOI:** 10.3390/ma15228120

**Published:** 2022-11-16

**Authors:** Lyudmila V. Parfenova, Zulfiya R. Galimshina, Guzel U. Gil’fanova, Eliza I. Alibaeva, Ksenia V. Danilko, Veta R. Aubakirova, Ruzil G. Farrakhov, Evgeny V. Parfenov, Ruslan Z. Valiev

**Affiliations:** 1 Institute of Petrochemistry and Catalysis, Ufa Federal Research Center, Russian Academy of Sciences, 141, Prospekt Oktyabrya, 450075 Ufa, Russia; 2 Central Research Laboratory, Bashkir State Medical University, 3 Lenin Street, 450000 Ufa, Russia; 3 Department of Electronic Engineering, Ufa State Aviation Technical University, 12 Karl Marx Street, 450008 Ufa, Russia; 4 Department of Materials Science and Physics of Metals, Ufa State Aviation Technical University, 12 Karl Marx Street, 450008 Ufa, Russia

**Keywords:** titanium implants, biocompatible coatings, c(RGDfC) peptide, bisphosphonic acids, nanostructured titanium, plasma electrolytic oxidation, in vitro tests, mesenchymal stem cells, fibroblasts, MG-63

## Abstract

Titanium is considered to be the most essential metal in the field of implantology. The main factors determining metal biocompatibility, among others, include the morphology and chemical composition of the titanium surface. Therefore, the aim of this work was to develop approaches to control the biological activity of the titanium surface by creating coatings that combine both an inorganic phase with a given morphology and organic molecules containing an integrin-selective peptide that regulate cell adhesion and proliferation. As such, we synthesized new c(RGDfC) derivatives of amino acid bisphosphonates (four examples) with different bisphosphonate anchors and maleimide linkers. These molecules were deposited on a highly developed porous surface obtained via the plasma electrolytic oxidation (PEO) of coarse-grained and nanostructured titanium. In vitro studies demonstrated the increase in the viability degree of mesenchymal stem cells and fibroblasts on the surface of coarse-grained or nanostructured titanium modified with PEO and a c(RGDfC) derivative of ε-aminocaproic acid bisphophonate with an SMCC linker. As a result, the use of conjugates of amino acid bisphosphonates with a cyclic RGD peptide for the modification of PEO-coated titanium opens the ways for the effective control of the biological activity of the metal implant surface.

## 1. Introduction

Titanium currently retains its position as the most commonly used metal in implantology [1]. Its excellent mechanical strength, chemical stability, and good biocompatibility predetermined the success of its widespread use of titanium and its alloys in this field of medicine. Recent developments led to the application of nanostructured titanium for dental and maxillofacial implants because of its higher strength and biocompatibility compared to commercially available Ti Grade 4 [2]. The quality of a titanium implant surface is one of the key points that contribute to the long-term clinical success of metal device implantation into a bone tissue. Despite the high biocompatibility of titanium, there is a need to solve the problem of a foreign body reaction, which triggers a cascade of biochemical processes preventing successful osseointegration and occasionally causing the device to be rejected [3]. Therefore, the development of methods for modifying the surface of implants by creating coatings that can provide better biocompatibility and control the response of the bone tissue is an important and topical problem. Moreover, the application of functional coatings on titanium implant surfaces moves the devices to a new generation, because the surface functionalization opens the possibilities to design a biomimetic surface that actively interacts with the extracellular matrix (ECM) [3]. Such a surface requires a coating with physical, chemical, and biological properties that are similar to those of bone; therefore, an inorganic layer with a morphology and phase composition similar to human bone should be used as a substrate in investigations into biologically active functional organic molecules. Such an inorganic layer can be formed on a titanium surface using the plasma electrolytic oxidation technique that has proven itself as an effective method for the development of biocompatible coatings on Ti alloys [4].

To create biologically active coatings, both short peptide sequences and common extracellular matrix proteins (for example, collagen, laminin, fibronectin, and osteopontin) are used. However, regulating the orientation and conformation of adsorbed and grafted large proteins, as well as attaining an appropriate amount of them for effective coating, is a non-trivial task [3]. Moreover, the use of high-molecular-weight ECM proteins is limited due to immunogenicity, difficulty in purification and manipulation, and the risk of pathogen transfer [5]. Therefore, the use of relatively short peptides seems to be more promising. In the mid-1980s, it was shown that many ECM proteins contain an amino acid sequence: arginine–glycine–aspartate (RGD), which binds with the cell surface specific receptors [6]. This discovery preceded the identification of a large group of adhesion receptors, so-called integrins, which specifically recognize the RGD fragment in particular ECM proteins. Integrins interact with ligands and activate intracellular signal transduction pathways serving as an intermediate link in cell migration and adhesion [7]. Integrin functioning significantly affects embryogenesis, angiogenesis, immune system, and tissue development [8].

Currently, there are dozens of short peptide sequences that interact in known ways with various cells. Among them, RGD is most often used for titanium surface modification [9,10,11,12,13,14,15,16]. Other examples of the use of various peptide sequences in combination with antifouling coatings to modify the implant surface are known [17,18,19].

To modify the surface, along with the use of the linear RGD molecule, the cyclic form of the oligopeptide, as well as the functionalized derivatives of the cyclic RGD fragment (N-methylated and N-acetylated c(RGDfV), phenyl-substituted c(RGD-b-HPhe), c(phg-isoDGR-G), etc.) can be applied [20], since cyclic RGD derivatives possess a number of advantages compared to the linear form: they have a higher selectivity for integrins of a certain type (for example, αvβ3 and α5β1) as well as metabolic and enzymatic stability [20,21,22,23,24,25,26,27,28]. Cyclic peptides c(RGDfK) [29,30,31,32,33,34,35,36,37,38,39] and c(RGDfV) [24,40,41] have previously been used to functionalize the titanium surface to improve osteosynthesis. In some works, a significant acceleration of osteosynthesis and a reduction in the fibrous process along the perimeter of the device in vivo were noted. However, there are rare examples in the literature on the use of cyclic tripeptide c(RGDfC) for the surface modification of titanium implants [41,42,43], although it is an attractive ligand due to cysteine ending, which can be easily involved in thiol-ene conjugation. Moreover, the cyclic peptide c(RGDfC) is a selective ligand for the ανβ3 integrin [44,45], which plays a significant role in bone tissue biology [46,47].

In our recent study, we carried out the synthesis of linear RGDC peptide conjugates with bisphosphonates of β-alanine, γ-aminobutyric, and ε-aminocaproic acids, which were used as the organic coatings for titanium with the surface modified by plasma electrolytic oxidation (PEO) [48,49,50]. The presence of gembisphosphonate groups in the molecule increases the degree of their affinity for the oxidized metal surface and gives better solubility in water [51,52]. In vitro studies have shown the promise of these molecules for use as coatings that enhance cell proliferation on the metal surface [48,49,50].

In continuation of these studies, taking into account the selectivity of cyclic forms of RGD peptide towards integrins and their high enzymatic stability, the aim of this research was to obtain bisphosphonate derivatives and to reveal their ability to influence the adhesion and viability of cells of various types on the metal surface comprising the effect of the substrate material—commercially available Ti Grade 4 and nanostructured Ti—on the surface’s biological properties.

## 2. Materials and Methods

### 2.1. Experimental Design

**Figure 1:**
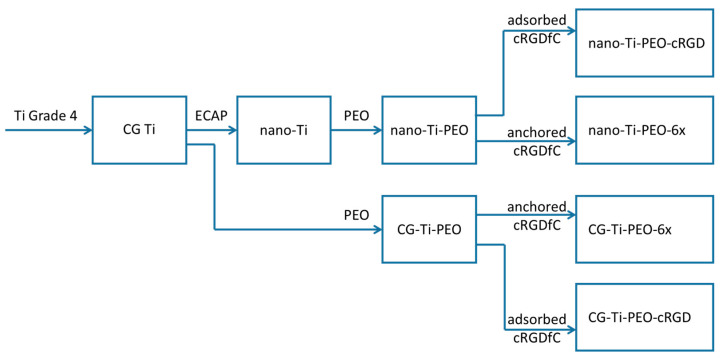
Experimental design scheme showing the steps of the sample preparation.

The experimental design is shown in Figure 1 as a sequence of the sample preparation steps. The experimental design operates with the following factors: substrate structure (2 levels—coarse grained (CG-Ti) commercially available Grade 4 and its nanostructured modification (nano-Ti)), the type of cRGDfC application to the surface (2 levels—adsorbed and anchored), and the structure of compound **6** (4 levels—**6a**, **6b**, **6c,** and **6d**) for the anchored cRGDfC. In total, this gives a fractional factorial design with 10 combinations.

In detail, the material treatments, sample preparation, and the surface modifications are described below.

### 2.2. Preparation of Titanium Samples and PEO Coating

Titanium Grade 4 (ASTM F67, chemical composition (wt.%): Fe—0.15, C—0.05, O—0.36, N—0.007; H—0.002, Ti—the rest) was used as the substrate material in this work. Titanium nanostructuring (nano-Ti) was carried out by means of equal-channel angular pressing (ECAP) followed by drawing [53]. As a result of severe plastic deformation, titanium rods with a diameter of 8 mm were obtained. Then, discs 0.5 mm-thick were cut from nano-Ti and CG-Ti rods using spark-cutting EDM equipment. Before the PEO treatment, they were ground by processing on SiC abrasive paper with a grit of 600, 1000, and 2000 to a roughness value of Ra < 0.15 µm. Afterwards, the samples were washed in distilled water and cleaned for 5 min in isopropyl alcohol using an ultrasound bath, before being dried in air at ambient conditions.

Both nano-Ti and CG-Ti sample disks were coated by the plasma electrolytic oxidation method in the same processing regime. The PEO coating process was carried out on an automated unit with a capacity of 50 kW, with the program control of the process parameters and maintaining them at a certain level with high accuracy.

Titanium alloy wire (Grade 2) with a diameter of 1 mm was applied as a holder. The titanium disk was placed in an 8 mm loop made at the end of the holder. The current supply was passed along the sample perimeter; the holder itself was also oxidized.

A glass vessel (10 L) equipped with a stainless steel heat exchanger arranged inside the electrolyte (5 L) around the walls was used for the plasma electrolytic oxidation. The PEO processing regime parameters are summarized in Table 1.

### 2.3. Synthesis of Conjugates of cRGD with Amino Acid Bisphosphonates

General Information. The following reagents were used for the synthesis: PCl_3’_ (98%, Acros Organics, Geel, Belgium), methanesulfonic acid (98%, Acros Organics), maleic anhydride (98+%, Acros Organics), β-alanine (97%, Acros Organics), γ-aminobutanoic (99+%, Acros Organics), ε-aminocaproic acid (98.5%, Merk, Darmstadt, Germany), dicyclohexylcarbodiimide (DCC, 99%, Acros Organics), N-hydroxysuccinimide (98+%, Acros Organics), and c(RGDfC) oligopeptide ((Cyclo(-Arg-Gly-Asp-D-Phe-Cys) acetate salt, Bachem, Bubendorf, Switzerland). The N- maleimidosuccinimide linkers (BMPS, EMCS, and SMCC) were obtained according to Refs. [54,55]. The synthesis of maleimides of γ-aminobutyric and ε-aminocaproic acid bisphosphonates **5a**–**d** were carried out according to [49].

Studies of the compound were carried out by means of one- ^1^H, ^13^C, ^31^P, and two-dimensional NMR (COSY ^1^H–^1^H, HSQC ^1^H–^13^C, HMBC ^1^H–^13^C) on a Bruker Avance II 500 HD Ascend spectrometer (operating frequency 500.17 MHz (^1^H), 125.78 MHz (^13^C), and 202.48 MHz (^31^P)). All the experiments were set up with standard Bruker pulse sequences. D_2_O was used as the solvent and internal standard. NMR ^31^P chemical shifts are given relative to the standard 85% solution of H_3_PO_4_ in H_2_O (δ_P_ 0 ppm). The chemical shifts of hydrogen atoms are given in the δ-scale (ppm) with respect to tetramethylsilane (TMS).

General procedure for the synthesis of c(RGDfC) conjugates. The cyclic peptide c(RGDfC) (3 mg, 0.005 mmol) was dissolved in 0.5 mL of bidistilled water, and the pH was adjusted to 7 by adding 0.1 N solution of NaOH. An equivalent amount of compounds **5a**–**d** (0.005 mmol) dissolved in 0.6 mL of water was added to the solution. The reaction mass was stirred for 1–2 h at 38–40 °C, then the solvent was evaporated under reduced pressure. Compounds **6a**–**d** were obtained as a white powder in 87–98% yields.

c(RGDfC)–BMPS–γ (**6a**). Yield 4.9 mg (98%). ^1^H NMR (D_2_O) δ: 1.41–1.57 (m, 2H, C^25^H_2_), 1.61–1.76 (m, 3H, C^3^H_2_, C^24^H), 1.78–1.90 (m, 3H, C^2^H_2_, C^24^H), 2.31–2.50 (m, 3H, C^6^H_2_, C^18^H), 2.54–2.64 (m, 1H, C^18^H), 2.73–2.83 (m, 2H, C^10^H, C^11^H), 2.88–3.05 (m, 2H, C^14^H_2_), 3.03–3.39 (m, 6H, C^4^H_2,_ C^10^H, C^11^H, C^26^H_2_), 3.44 and 4.12 (both d, ^2^*J* = 15.0 Hz, 2H, C^21^H_2_), 3.64–3.78 (m, 2H, C^7^H_2_), 4.20–4.39 (m, 3H, C^9^H, C^12^H, C^23^H), 4.57–4.65 (m, 2H, C^15^H, C^17^H,), 7.15–7.25 (m, 3H, Ph c(RGDfC)), 7.25–7.33 (m, 2H, Ph c(RGDfC)). ^13^C NMR (D_2_O) δ: 23.59 (C^3^), 24.51 (C^25^), 26.92 (C^24^), 31.42 (C^11^), 31.39 (C^2^), 31.88 (C^11^), 32.84 (C^10^), 35.27 (C^6^), 35.78 (C^7^), 36.32 (C^14^), 37.88 (C^18^), 40.51 (C^4^,C^26^), 43.57 (C^21^), 50.84 (C^17^), 52.74 (C^23^), 54.04 (C^12^), 54.42 (C^9^), 55.10 (C^15^), 73.83 (C^1^), 127.12 (Ph c(RGDfC)), 128.75 (Ph c(RGDfC)), 129.22 (Ph c(RGDfC)), 136.10 ((Ph c(RGDfC)), 156.65 (C^27^), 171.24 (C^16^), 172.16 (C^28^), 172.66 (C^20^, C^5^), 172.67 (C^22^), 173.61 (C^13^), 177.97 (C^19^). ^31^P NMR (D_2_O) δ: 18.37.

c(RGDfC)–EMCS–ε (**6b**). Yield 4.8 mg (89%). ^1^H NMR (D_2_O) δ: 1.12–1.31 (m, 4H, C^4^H_2_, C^10^H_2_), 1.38–1.57 (m, 10H, C^3^H_2_, C^5^H_2_, C^9^H_2_, C^11^H_2_, C^30^H_2_), 1.58–1.72 (m, 1H, C^29^H), 1.74–1.90 (m, 3H, C^2^H_2_, C^29^H), 2.09–2.19 (m, 2H, C^8^H_2_), 2.39–2.49 (m, 1H, C^23^H), 2.50–2.63 (m, 1H, C^23^H), 2.74–2.82 (m, 2H, C^14^H, C^16^H), 2.88–3.06 (m, 2H, C^20^H_2_), 3.02–3.12 (m, 2H, C^14^H, C^16^H), 3.05–3.19 (m, 4H, C^6^H_2_, C^31^H_2_), 3.36–3.51 (m, 3H, C^26^H, C^12^H_2_), 4.13 (d, ^2^*J* = 15.0 Hz, 1H, C^26^H), 4.20–4.30 (m, 1H, C^17^H), 4.30 (dd, ^3^*J* = 9.50 Hz, ^3^*J* = 5.50 Hz, 1H, C^28^H), 4.33–4.41 (m, 1H, C^15^H), 4.46–4.64 (m, 2H, C^19^H, C^22^H), 7.15–7.24 (m, 3H, Ph c(RGDfC)), 7.26–7.33 (m, 2H, Ph c(RGDfC)). ^13^C NMR (D_2_O) δ: 24.75 (C^3^, C^9^, C^11^), 25.21 (C^10^), 27.04 (C^29^, C^4^), 28.20 (C^5^, C^30^), 32.45 (C^16^), 33.78 (C^2^), 35.56 (C^8^), 36.27 (C^20^), 37.89 (C^23^), 38.92 (C^12^), 39.41 (C^6^, C^31^), 41.10 (C^14^), 43.73 (C^26^), 50.84 (C^22^), 52.81 (C^28^), 53.80 (C^17^), 54.98 (C^15^), 55.62 (C^19^), 127.05 (Ph c(RGDfC)), 128.86 (Ph c(RGDfC)), 129.23 (Ph c(RGDfC)), 136.11 (Ph c(RGDfC)), 171.78 (C^25^), 172.50 (C^21^), 172.65 (C^33^), 172.83 (C^18^, C^27^), 176.80 (C^7^), 177.87 (C^24^). ^31^P NMR (D_2_O) δ: 18.68.

c(RGDfC)–SMCC–γ (**6c**). Yield 4.9 mg (91%). ^1^H NMR (D_2_O) δ: 0.87–1.01 (m, 2H, C^8^H_2_), 1.19–1.34 (m, 2H, C^7^H_2_), 1.42–1.56 (m, 2H, C^28^H_2_) 1.56–1.70 (m, 4H, C^8^H_2_, C^9^H, C^27^H), 1.67–1.97 (m, 7H, C^2^H_2_, C^3^H_2_, C^7^H_2_, C^27^H), 2.05–2.16 (m, 1H, C^6^H), 2.44 (dd, ^3^*J* = 7.5 Hz, ^2^*J* = 15.0 Hz, 1H, C^21^H), 2.52–2.61 (m, 1H, C^21^H), 2.73 (dd, ^3^*J* = 5.0 Hz, ^2^*J* = 14.0 Hz, 1H, C^12^H), 2.78 (dd, ^3^*J* = 9.5 Hz, ^2^*J* = 14.0 Hz, 1H, C^14^H), 2.90–3.06 (m, 5H, C^4^H_2_, C^14^H, C^18^H_2_), 3.07–3.16 (m, 3H, C^12^H, C^29^H_2_), 3.24–3.36 (m, 2H, C^10^H_2_), 3.41 and 4.14 (both d, ^2^*J* = 14.8 Hz, 2H, C^24^H_2_), 4.25 (dd, ^3^*J* = 5.0 Hz, ^3^*J* = 9.0 Hz, 1H, C^15^H), 4.26–4.41 (m, 1H, C^26^H), 4.38–4.47 (m, 1H, C^13^H), 4.51–4.63 (m, 2H, C^17^H, C^20^H), 7.15–7.24 (m, 3H, Ph c(RGDfC)), 7.26–7.33 (m, 2H, Ph c(RGDfC)). ^13^C NMR (D_2_O) δ: 23.48 (C^3^), 24.45 (C^28^), 27.03 (C^27^), 28.32 (C^7^), 29.13 (C^8^), 31.18 (C^2^), 31.70 (C^14^), 33.24 (C^12^), 35.40 (C^9^), 36.29 (C^18^), 37.87 (C^21^), 40.49 (C^4^, C^29^), 43.68 (C^24^), 44.59 (C^6^), 44.88 (C^10^), 50.83 (C^20^), 52.74 (C^26^), 54.03 (C^15^), 54.55 (C^13^), 55.43 (C^17^), 127.12 (Ph c(RGDfC)), 128.86 (Ph c(RGDfC)), 129.26 (Ph c(RGDfC)), 136.02 (Ph c(RGDfC)), 156.66 (C^30^), 171.26 (C^23^), 172.48 (C^19^, C^25^), 172.61 (C^31^), 177.86 (C^22^). ^31^P NMR (D_2_O) δ: 18.27.

c(RGDfC)–SMCC–ε (**6d**). Yield 4.8 mg (87%). ^1^H NMR (D_2_O) δ: 0.87–1.03 (m, 2H, C^10^H_2_), 1.19–1.39 (m, 4H, C^9^H_2_, C^4^H_2_), 1.42–1.51 (m, 2H, C^5^H_2_), 1.50–1.57 (m, 2H, C^3^H_2_), 1.39–1.52 (m, 2H, C^30^H_2_), 1.51–1.64 (m, 1H, C^11^H), 1.57–1.70 (m, 4H, C^10^H_2_, C^29^H_2_), 1.70–1.79 (m, 2H, C^9^H_2_), 1.76–1.88 (m, 1H, C^29^H), 1.79–1.90 (m, 2H, C^2^H_2_), 2.03–2.17 (m, 1H, C^8^H), 2.45 (dd, ^2^*J* = 15.8 Hz, ^3^*J* = 7.3 Hz, 1H, C^23^H), 2.57 (dd, ^2^*J* = 15.8 Hz, ^3^*J* = 7.0 Hz, 1H, C^23^H), 2.58–2.69 (m, 2H, C^16^H_2_), 2.94 (dd, ^2^*J* = 13.0 Hz, ^3^*J* = 10.0 Hz, 1H, C^20^H), 3.03 (dd, ^2^*J* = 13.0 Hz, ^3^*J* = 6.0 Hz, 1H, C^20^H), 3.04–3.19 (m, 4H, C^6^H_2_, C^31^H_2_), 3.26–3.33 (m, 2H, C^12^H_2_), 3.42 and 4.14 (both d, ^2^*J* = 14.8 Hz, 2H, C^26^H_2_), 4.12 (dd, ^3^*J* = 7.6 Hz, ^3^*J* = 5.2 Hz, 1H, C^17^H), 4.32 (dd, ^3^*J* = 8.5 Hz, ^3^*J* = 6.0 Hz, 1H, C^28^H), 4.55 (dd, ^3^*J* = 10.0 Hz, ^3^*J* = 6.0 Hz, 1H, C^19^H), 4.61 (t, ^3^*J* = 7.0 Hz, 1H, C^22^H), 7.15–7.24 (m, 3H, Ph c(RGDfC)), 7.26–7.33 (m, 2H, Ph c(RGDfC)). ^13^C NMR (D_2_O) δ: 23.40 (C^3^), 24.52 (C^16^), 26.94 (C^4^), 27.19 (C^29^), 28.29 (C^30^), 28.57 (C^5^), 28.59 (C^9^), 29.17 (C^10^), 33.74 (C^2^), 37.87 (C^23^), 39.42 (C^6^), 40.51 (C^31^), 43.70 (C^26^), 44.73 (C^12^), 44.76 (C^8^), 50.85 (C^22^), 52.72 (C^28^), 55.43 (C^19^), 127.25 (Ph c(RGDfC)), 128.87 (Ph c(RGDfC)), 129.22 (Ph c(RGDfC)), 136.03 (Ph c(RGDfC)), 156.60 (C^32^), 171.21 (C^33^), 172.08 (C^25^), 172.49 (C^21^), 172.69 (C^27^), 173.61 (C^18^), 177.88 (C^24^). ^31^P NMR (D_2_O) δ: 18.62.

### 2.4. Surface Characterization

The surface morphology of the PEO-coated samples as substrates for the organic coating application was assessed using the Regulus 8220 (Hitachi, Tokyo, Japan) scanning electron microscope (SEM). The visible (surface) coating porosity and average pore size were calculated with ImageJ software from the SEM images according to ASTM E112–10 standard. The PEO coating thickness was measured using Positector 6000 (Defelsko, Ogdensburg, NY, USA) eddy current sensor with N-type gauge. The surface roughness parameters Ra and Rz were measured using TR-220 profilometer (Time High Technology, Beijing, China). The phase constituents of the PEO coating were assessed with X-ray diffractometer Ultima IV, (Rigaku, Tokyo, Japan). The resultant diffractograms were evaluated using Philips XPert software (Philips, Amsterdam, Netherlands); for the quantitative analysis, a semiquant algorithm embedded into the software was employed.

To characterize the organic part of the coating, X-ray photoelectron spectroscopy (XPS) was carried out using a JEOL JPS 9010MX spectrometer equipped with an (Mg Kα) X-ray source. The spectra were acquired at the pressure less than 7 × 10^−8^ Pa in the analytical chamber. The survey spectra were collected from 0 to 1000 eV with a pass energy of 50 eV, and high-resolution spectra were collected for each detected element of interest (C, N, O, P, S, and Ti) with a pass energy of 10 eV. For the characteristic peak identification, the elemental composition calculations, and the peak fitting in the high-resolution spectra the JEOL SpecSurf Program V. 1.9.2 was used.

### 2.5. Response of Human Cells to Conjugates of c(RGDfC) with Amino Acid Bisphosphonates and Ti-PEO Coating

Human osteosarcoma cells (MG-63) were obtained from the Russian collection of cell cultures of the Institute of Cytology, Russian Academy of Sciences (Institute of Cytology, St. Petersburg, Russia). Human adipose tissue mesenchymal stem cells (MSCs) and embryonic human lung fibroblasts (FLECH-104) (BIOLOT, St. Petersburg, Russia) were used for in vitro studies as well. MG-63 and FLECH-104 cells were cultured in Dulbecco’s modified Eagle medium (DMEM) (Sigma-Aldrich, Burlington, MA, USA) containing 10% fetal bovine serum (FBS) (BioWest, Nuaillé, France). MSCs were cultured in DMEM with 20% FBS and 1 g/L glucose (Sigma) in a humidified atmosphere with 5% CO_2_ in 25 cm^2^ culture flasks (SPL Life Sciences, Pocheon-si, Republic of Korea). The culture medium was changed twice a week. Following monolayer formation, cells were separated using 0.25% trypsin solution (PANECO, Moscow, Russia) and counted by an automatic cell counter TC20 (BioRad, Hercules, CA, USA) and used for further experiments.

The MTT test was used to evaluate the cell viability in culture medium with c(RGDfC) its derivatives **6a–d**, which assesses the cell metabolic activity proportional to their number. MSCs (5 × 10^3^) were seeded into a 96-well culture plate, and when they reached a monolayer, 5 vol% solutions of organic compounds (0.1–0.01 mg/mL) were added to the complete medium, 6 wells of each type were used. Culture medium with only solvents of organic compounds was used as cell viability control. Cells were incubated with organic compounds under standard conditions (5 vol% CO_2_, 37 °C) within 7 days. MSCs in complete culture medium with 5% of the solvent for organic derivatives served as controls. The medium was substituted with an MTT solution (0.5 mg/mL) for 3.5 h, and then with dimethyl sulfoxide to dissolve the formazan crystals formed in the mixture. The plate reader Spark10M (Tecan, Männedorf, Switzerland) was applied to measure the absorbance at 530 nm with a reference wavelength of 620 nm.

A modification of the MTT test, EZ4U (Biomedica), was used to analyze cell proliferation. Ti samples with PEO coating were cleaned in 95% ethanol for 10 min in ultrasonic bath and then washed with deionized water, dried in air atmosphere and autoclaved at 134 °C. For the deposition of organic molecules on the surface of PEO-coated Ti disks, these were kept in a sterile 15 mL centrifuge tube with solutions of organic derivatives in a solvent system of ethanol–DMSO at a ratio of 100:9 with a substance content of 0.1 mg/mL for 1 h. The solutions were pre-sterilized by filtration through the 0.22 μm CA filter. Then, the samples were dried in air and placed in a 48-well culture plate. A suspension of MSC, FLECH-104, or MG-63 cells was placed in the plate (0.4 mL, containing (6–10) × 10^3^ cells) and incubated under standard conditions for 7 days. The number of cells was compared with the control uncoated Ti-PEO samples. The culture plate well (polystyrene) served as the background. After incubation, the samples of each type were transferred to another 48-well plate loaded with 0.4 mL of fresh complete medium. Then, activated EZ4U solution (40 µL) was added to each well and incubated for 3.5 h at 37 °C, 5 vol% CO_2_. The optical absorbance of the solution was analyzed at 450 nm with a reference wavelength of 620 nm. The percentage of EZ4U metabolization was normalized with respect to the Ti-PEO control group.

The mean value and the standard deviation for 6 (MTT) and 3 (EZ4U) optical density measurements were calculated relative to the corresponding control. As the number of repeats was less than 10, we used the Kruskal–Wallis test with subsequent Dunn’s multiple comparisons test (GraphPad Prism, v.6.01) at a significance level of *p* < 0.05 was used for data analysis.

## 3. Results and Discussions

### 3.1. Surface Properties of the PEO-Coated Ti Disks as Substrates for the cRGDfC Assessment

**Figure 2:**
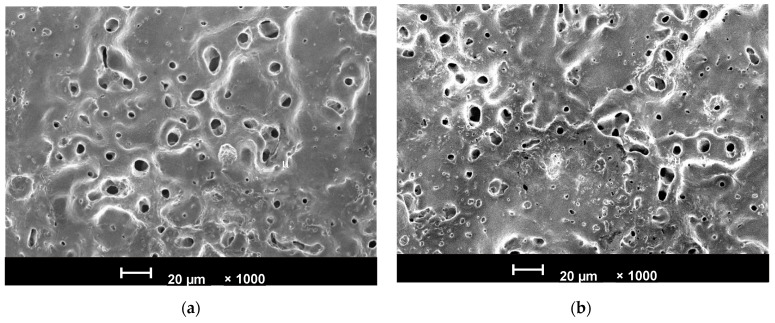
SEM images of the top view of the PEO coatings on CG-Ti (**a**), nano-Ti (**b**).

The PEO coating on CG-Ti and nano-Ti has a porous structure which is beneficial for the cell fixation, as shown elsewhere [7]. As seen from Figure 2 and Table 2, the coating morphology slightly differs between CG-Ti and nano-Ti. The coating on nano-Ti is 15% thicker, so the roughness Rz appears also higher. The coating porosity value is similar for the both substrates; however, the pore size distribution is different: the PEO coating on nano-Ti (Figure 2a) has numerous smaller pores. As a result, the average pore size is twice lower than that for the CG-Ti. This is consistent with earlier studies [48,56]. Since the pore size affects the ability of cells to attach to the surface, it is expected that these two substrates have different cell responses. As follows from the results of the X-ray diffractometry (Table 2, Figure S1—Supplementary material), the main crystalline constituent of the PEO coating is anatase, which is a lower temperature phase of the titania, and the anatase is more biocompatible than rutile [57]. Larger pores in the PEO coating appear due to larger microdischarges during the process, thus resulting in higher rutile content for the CG-Ti substrate. This dependency was described earlier, and these results are consistent with the previous studies [58].

### 3.2. Synthesis and Biological Properties of the cRGDfC Organic Coatings

**Scheme 1:**
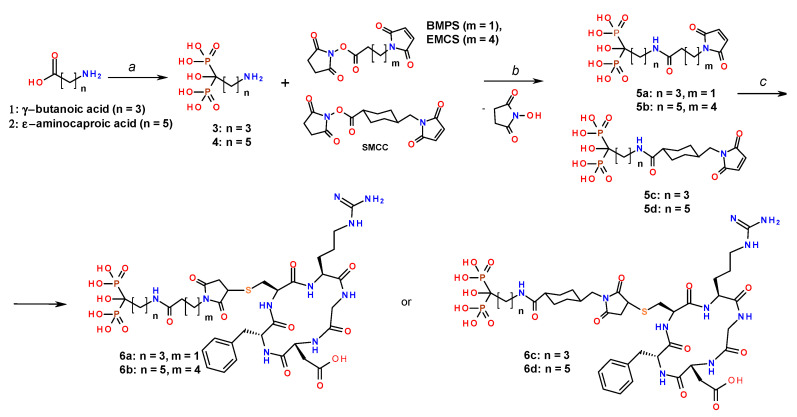
Reaction conditions: **a**. PCl_3_, MeSO_3_H, 85–90 °C, 4–5 h; **b**. H_2_O:acetone = 1:1, pH = 8–9, r.t., 1 h; **c**. c(RGDfC), H_2_O, pH = 7, 38–40 °C, 1–1.5 h.

We synthesized a set of c(RGDfC) (cyclo(-Arg-Gly-Asp-D-Phe-Cys)) derivatives, in which the peptide fragment was conjugated with γ-aminobutyric and ε-aminocaproic acid bisphosphonates via BMPS, EMSC, and SMCC linkers (Scheme 1). For this purpose, we preliminarily obtained N-maleimides of γ-butanoic and ε-caproic acids bisphosphonates (**5a**–**d**) according to our previously developed procedure [49]. The conjugation of compounds **5a**–**d** with c(RGDfC) was carried out via Michael addition in an aqueous medium at 38–40 °C, as a result of which compounds **6a**–**d** were observed (Scheme 1).

The structures of compounds **6a**–**d** were proven using one- (^1^H, ^13^C, ^31^P) and two-dimensional (COSY HH, HSQC, and HMBC) NMR spectroscopy. The ^1^H NMR spectra showed signals corresponding to c(RGDfC), linker, and aminobisphosphonate fragments (Supplementary material, Figures S2–S5). The only resonance lines at δ_P_ 17–18 ppm in the ^31^P NMR spectra indicated the presence of the bisphosphonate group in the molecules. The addition of c(RGDfC) to **5a–d** led to the disappearance of the signal at δ_H_ 6–7 ppm corresponding to the N-maleimide double bond fragment.

**Figure 3:**
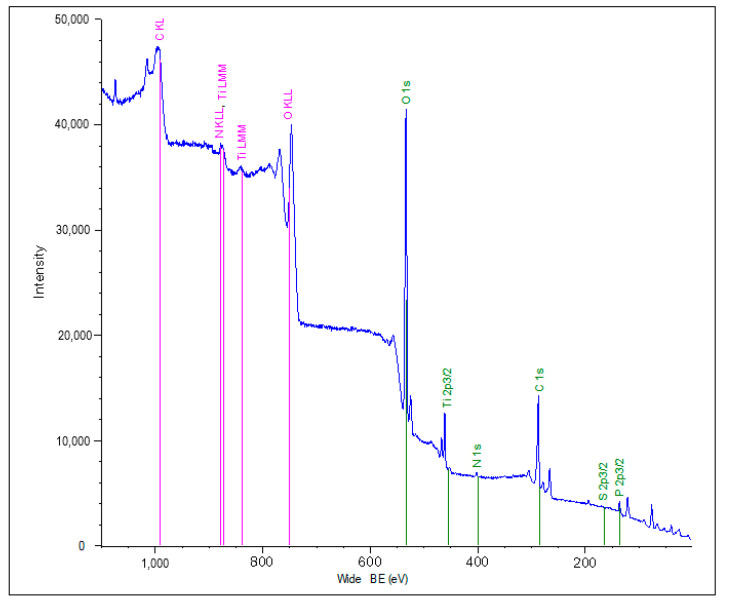
Survey XPS spectra of the CG-Ti-PEO-**6d** coating.

The occurrence of organic molecules in the porous PEO sublayer was established by X-ray photoelectron spectroscopy (XPS). Peaks of Ti2p, Ti3s, O1s, C1s, P2p, N1s, and S2s were observed in the XPS spectra (Figure 3). XPS analysis of the c(RGDfC)-modified samples showed changes in the chemical composition of the surface compared to the sample without an organic coating. The greatest differences were found for the Ti2p, C1s, O1s, and N1s peaks. The appearance of the organic compounds provided the decrease in the Ti2p line intensity and the increase in the C1s’, O1s’, and N1s’ intensity, which corresponded to a decrease in the Ti2p/C1s, Ti2p/O1s, and Ti2p/N1s ratios compared to the control (Table 3).

**Figure 4:**
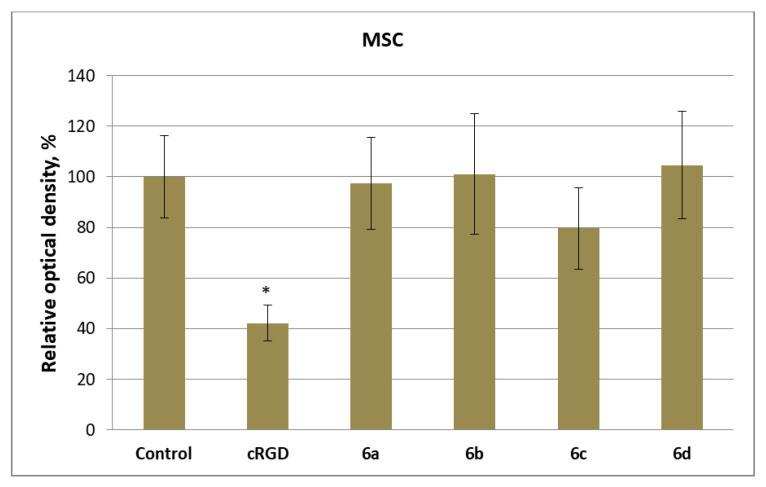
Cell viability established by the MTT test in the presence of compounds. Significant differences with the control (*p* < 0.05) are shown with asterisks.

**Figure 5:**
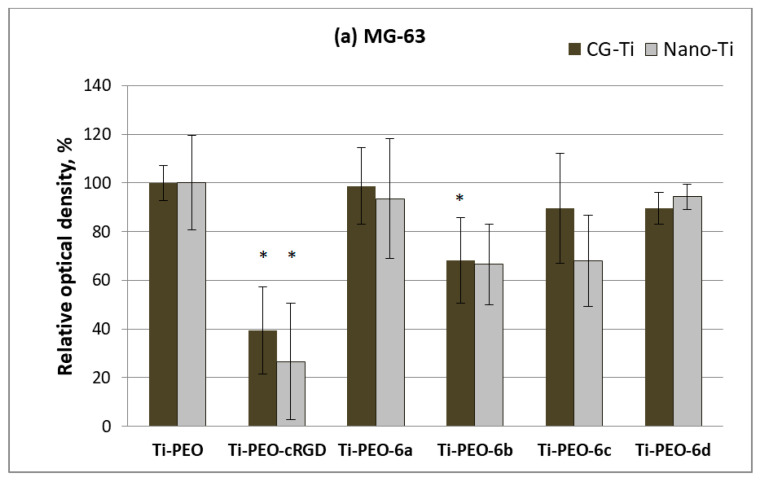

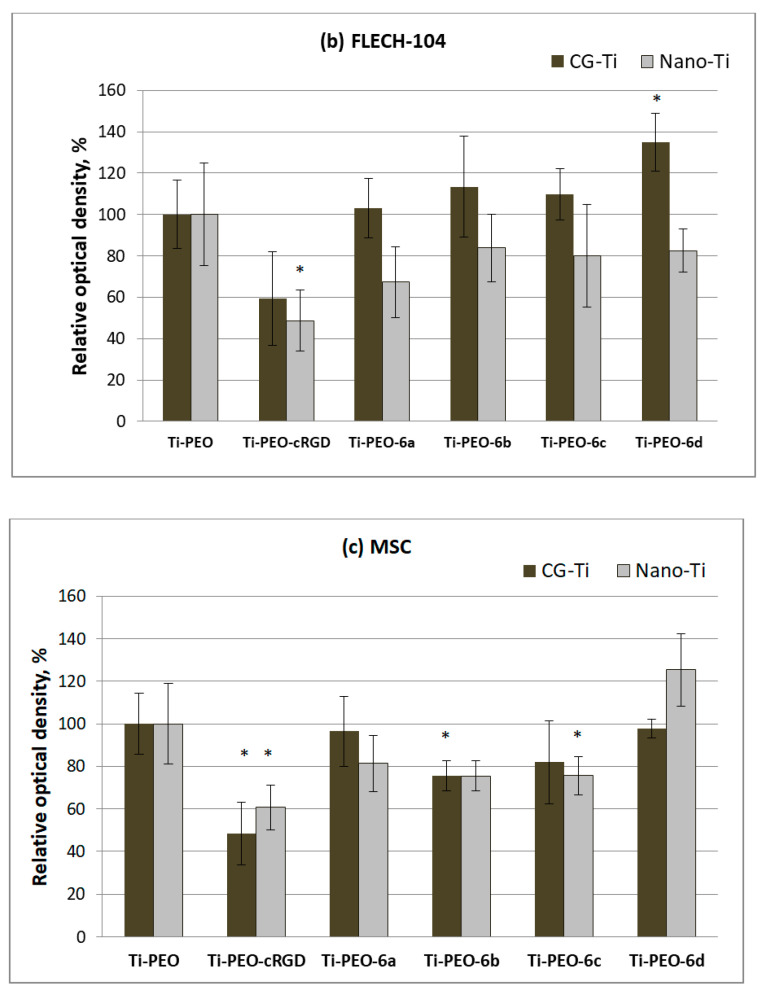
EZ4U in vitro assay of the growth of MG-63 osteoblasts (**a**), FLECH-104 fibroblasts (**b**), and adipose-derived MSCs (**c**) cultivated within 7 days on Ti-PEO samples coated with compounds c(RGDfC), **6a**–**d**. Significant differences with the control (*p* < 0.05) are shown with asterisks.

In vitro studies of the viability and proliferation of mesenchymal stem cells, fibroblasts, and osteoblast-like cells (MG-63) demonstrated the dependence of the biological activity on the structure of the molecules introduced into the PEO-modified surface of nano- and CG-Ti samples (Figure 4 and Figure 5a–c). The evaluation of the cell viability with c(RGDfC) and its derivatives **6a**–**d** in culture medium showed a decrease of up to 40% for the unmodified c(RGDfC) (Figure 4), while the appearance of a linker and a bisphosphonate group in the structure overcame this problem.

There is the same effect of the reduction in the degree of viability of all the studied cell types on the Ti-PEO surface containing the c(RGDfC) peptide (Figure 5a–c). The appearance of molecules **6b** and **6c** in the composition of the surface also reduced the proliferation of osteoblast-like cells MG-63 and MSCs. This effect is likely due to toxicity of RGD and some of its derivatives and may be related to the known effect of cell death via the apoptotic pathway as a result of exposure to these molecules [59,60].

Using the organic molecule **6d** with the SMCC linker and ε-aminocaproic acid bisphosphonate fragment (Ti-PEO-**6d**) in combination with PEO-modified coarse-grained Ti (Figure 5b) provided a significant (up to 30–40%) increase in the fibroblast proliferation. The same molecule caused 20% growth in MSCs on the surface of nanostructured titanium (Figure 5c).

## 4. Conclusions

New conjugates of the c(RGDfC) peptide with amino acid bisphosphonates were synthesized. Compounds can be introduced into the pores of the PEO-coated Ti via physicochemical adsorption from solutions.

In vitro studies on the proliferation and viability of fibroblasts, mesenchymal stem cells, and osteoblast-like cells have shown the dependence of the biological activity of molecules on its structure. It was shown that c(RGDfC) itself reduces cell viability, and it is apparently toxic. This effect most likely determined the decrease in the degree of proliferation of all cell types studied by us on the Ti surface containing the original peptide. The appearance of a linker and a bisphosphonate anchor in the structure reduced the toxicity of cyclo-RGD, but only the ε-aminocaproic acid derivative with an SMCC linker was able to increase the degree of fibroblasts and mesenchymal stem cell proliferation on the surface of CG or nanostructured titanium. Thus, the use of the integrin-active peptide of cyclic structure makes it possible to effectively control the biological activity of the titanium surface, and the structure of the bisphosphonate anchor and linker as well as the type of metal substrate is of great importance.

## Figures and Tables

**Table 1:** PEO processing regime.

Electrolyte Composition	Positive Pulse		Negative Pulse		Frequency (Hz)	Temperature (°C)	Duration (min)
	Voltage (V)	Duty Cycle (%)	Voltage (V)	Duty Cycle (%)			
20 g/L Na_3_PO_4_·12 H_2_O	470 ± 5	51 ± 0.1	40 ± 1	26 ± 0.1	300 ± 1	20 ± 1	5′ ± 5″

**Table 2:** Properties of the PEO coatings on coarse-grained and nanostructured Ti disks.

Sample	h (μm)	Rz (μm)	Ra (μm)	Porosity (%)	Average Pore Size (μm)	Anatase Content (%)	Rutile Content (%)
CG-Ti-PEO	18.9 ± 1.1	15.6 ± 0.54	2.6 ± 0.13	7.6 ± 1.4	1.44 ± 0.17	62 ± 2%	38 ± 2%
Nano-Ti-PEO	21.6 ± 1.3	17.3 ± 2.54	2.5 ± 0.05	7.6 ± 1.5	0.61 ± 0.11	70 ± 2%	30 ± 2%

**Table 3:** Atomic composition and ratio for the Ti-PEO samples obtained from XPS high-resolution spectra.

Sample		Atomic Composition (%)						Atomic Ratio		
Type of Organic Coating	Substrate	Ti2p	O1s	C1s	N1s	P2p	S2s	Ti2p/O1s	Ti2p/C1s	Ti2p/N1s
-	CG-Ti-PEO	11.70	60.70	20.45	0.79	2.47	0.00	0.19	0.57	14.81
	Nano-Ti-PEO	9.78	58.38	25.36	0.95	2.45	0.00	0.17	0.39	10.29
c(RGDfC)	CG-Ti-PEO	5.50	67.42	22.78	0.54	1.63	0.00	0.08	0.24	10.19
	Nano-Ti-PEO	4.07	76.25	17.19	0.40	0.57	0.00	0.05	0.24	10.18
**6a**	CG-Ti-PEO	0.89	76.00	21.90	0.26	0.78	0.00	0.01	0.04	3.42
	Nano-Ti-PEO	5.59	70.58	20.16	0.65	1.28	0.00	0.08	0.28	8.60
**6b**	CG-Ti-PEO	4.65	69.48	22.66	0.68	0.82	0.20	0.07	0.21	6.84
	Nano-Ti-PEO	3.46	68.09	23.27	0.55	0.47	0.31	0.05	0.14	6.29
**6c**	CG-Ti-PEO	9.08	70.59	14.44	0.71	2.01	0.11	0.13	0.63	12.79
	Nano-Ti-PEO	3.10	68.35	25.95	0.56	0.31	0.22	0.05	0.12	5.54
**6d**	CG-Ti-PEO	6.28	68.19	19.52	2.28	1.15	0.24	0.09	0.32	2.75
	Nano-Ti-PEO	6.39	68.66	20.26	0.65	1.32	0.00	0.09	0.32	9.83

## Data Availability

Not applicable.

## Supplementary Materials

The following supporting information can be downloaded at: https://www.mdpi.com/article/10.3390/ma15228120/s1, Figures S1–S5: XRD, ^1^H, ^13^C, ^31^P and COSY HH NMR of compounds **6a**–**d**.
