# Supplementary material for: Modeling of Biological Activity of PEO-Coated Titanium Implants with Conjugates of Cyclic RGD Peptide with Amino Acid Bisphosphonates
Source: Materials (Basel). 2022 Nov 16;15(22):8120. doi: 10.3390/ma15228120 (PMC9699121; doi:10.3390/ma15228120)

Modeling of biological activity of PEO-coated titanium implants with conjugates of cyclic RGD peptide with amino acid bisphosphonates

Lyudmila V. Parfenova[1], Zulfiya R. Galimshina[1], Guzel U. Gil'fanova[1], Eliza I. Alibaeva[1], Ksenia V. Danilko[2], Veta R. Aubakirova[3], Ruzil G. Farrakhov[3], Evgeny V. Parfenov[3], and Ruslan Z. Valiev[3]

[1]Institute of Petrochemistry and Catalysis, Ufa Federal Research Center, Russian Academy of Sciences, 141, Prospekt Oktyabrya, 450075 Ufa, Russia

[2]Bashkir State Medical University, 3 Lenin Street, 450000 Ufa, Russia

[3]Ufa State Aviation Technical University, 12 Karl Marx Street, 450008 Ufa, Russia

Supporting Information

Figure S1. XRD patterns of Ti and Ti-PEO with the labeled peaks...............2
Figure S2. ^{1}H, ^{13}C, ^{31}P and COSY HH NMR of compound (**6a**)....................3
Figure S3. ^{1}H, ^{13}C, ^{31}P and COSY HH NMR of compound (**6b**)....................5
Figure S4. ^{1}H, ^{13}C, ^{31}P and COSY HH NMR of compound (**6c**)....................8
Figure S5. ^{1}H, ^{13}C, ^{31}P and COSY HH NMR of compound (**6d**)..................12

Figure S1. XRD patterns of Ti and Ti-PEO with the labeled peaks.

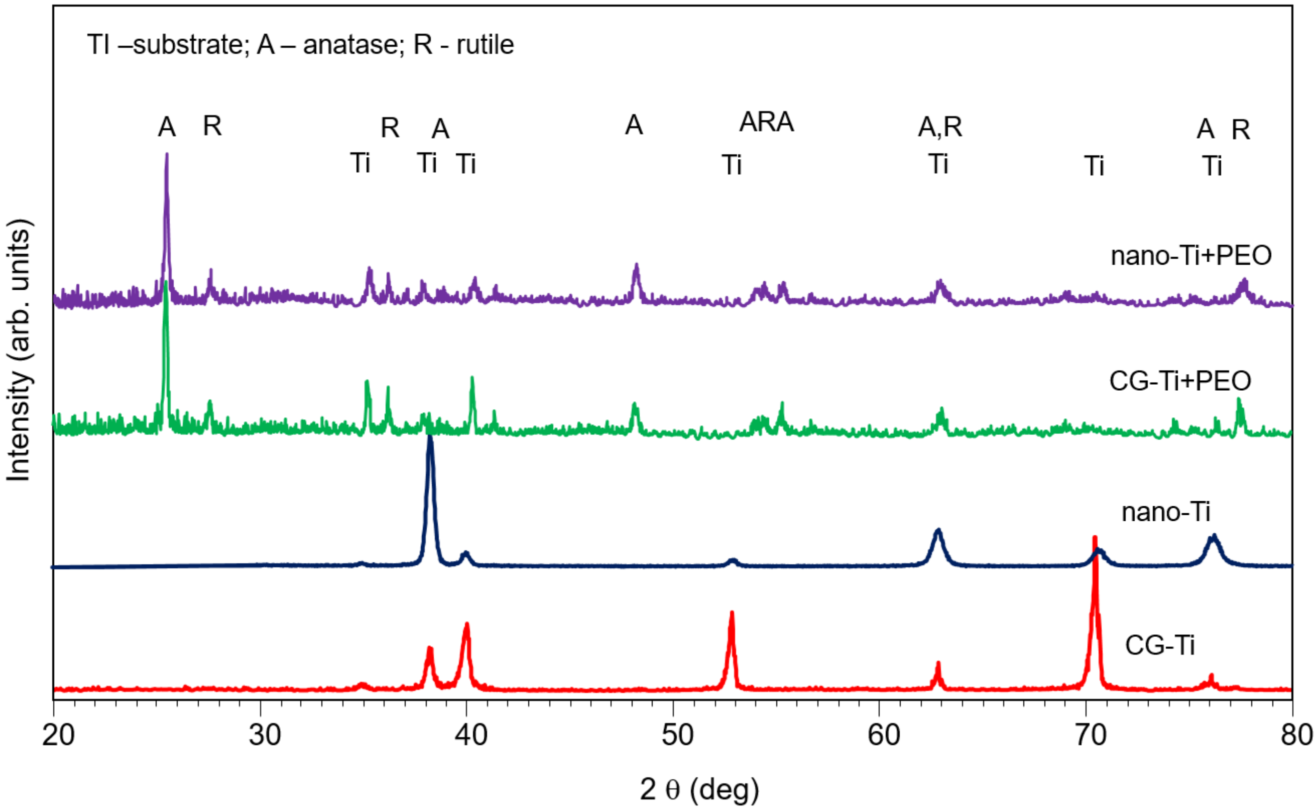

Figure S2. ^{1}H, ^{13}C, ^{31}P and COSY HH NMR of compound (**6a**).

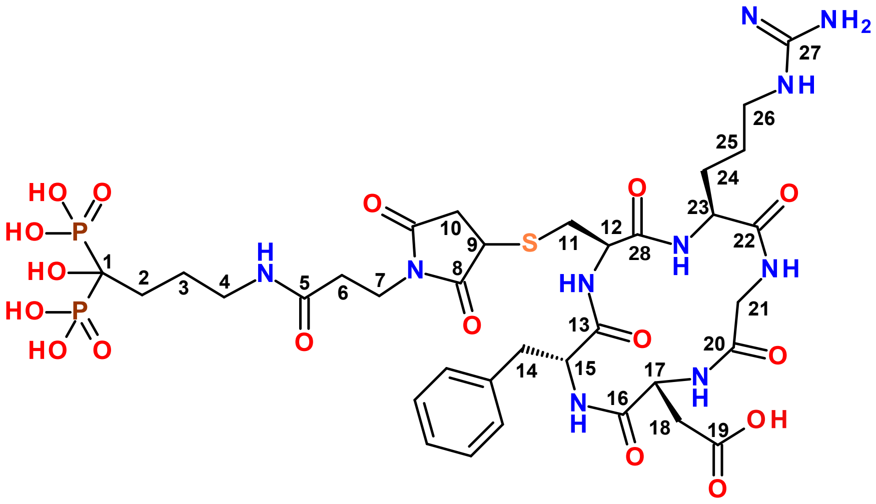

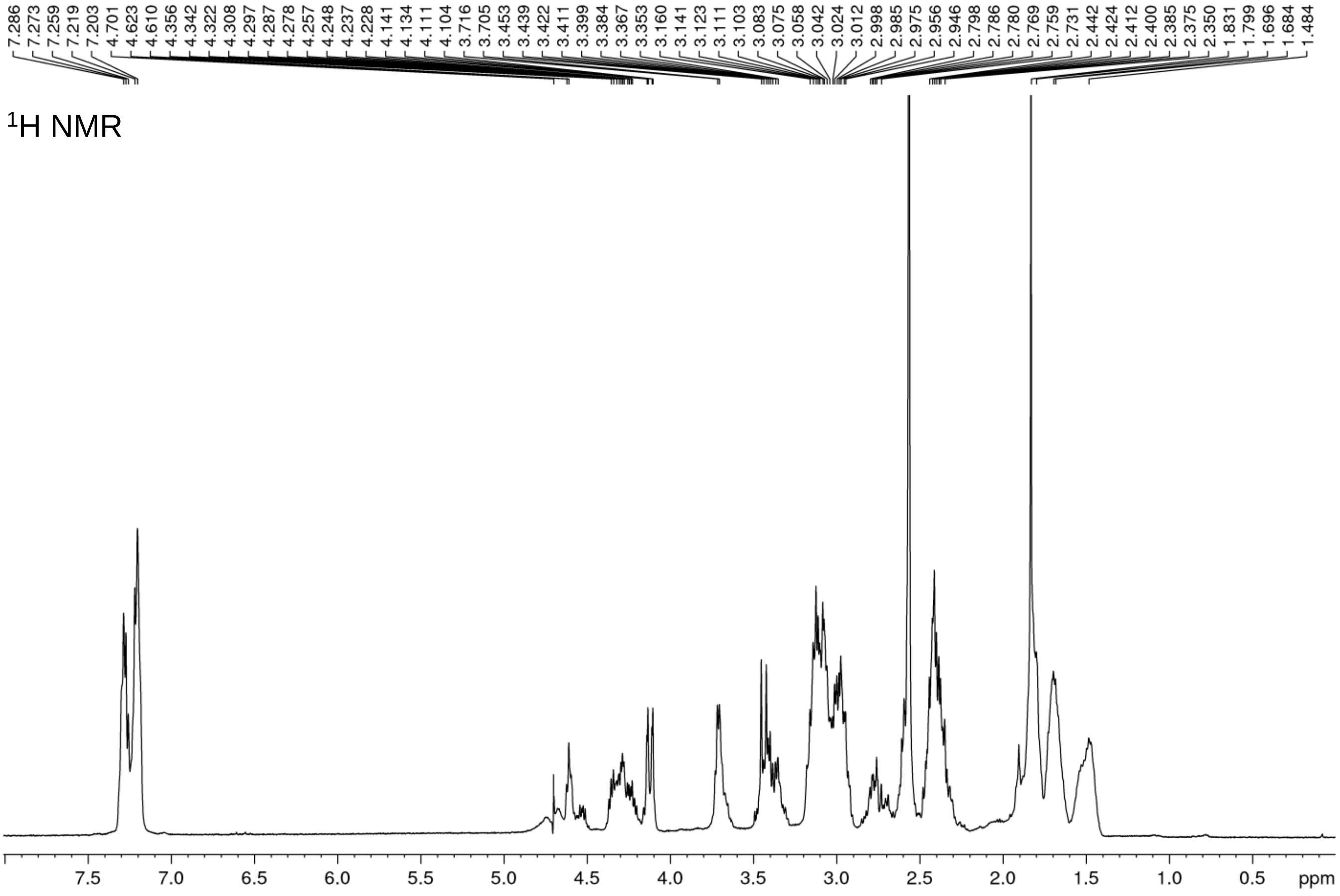

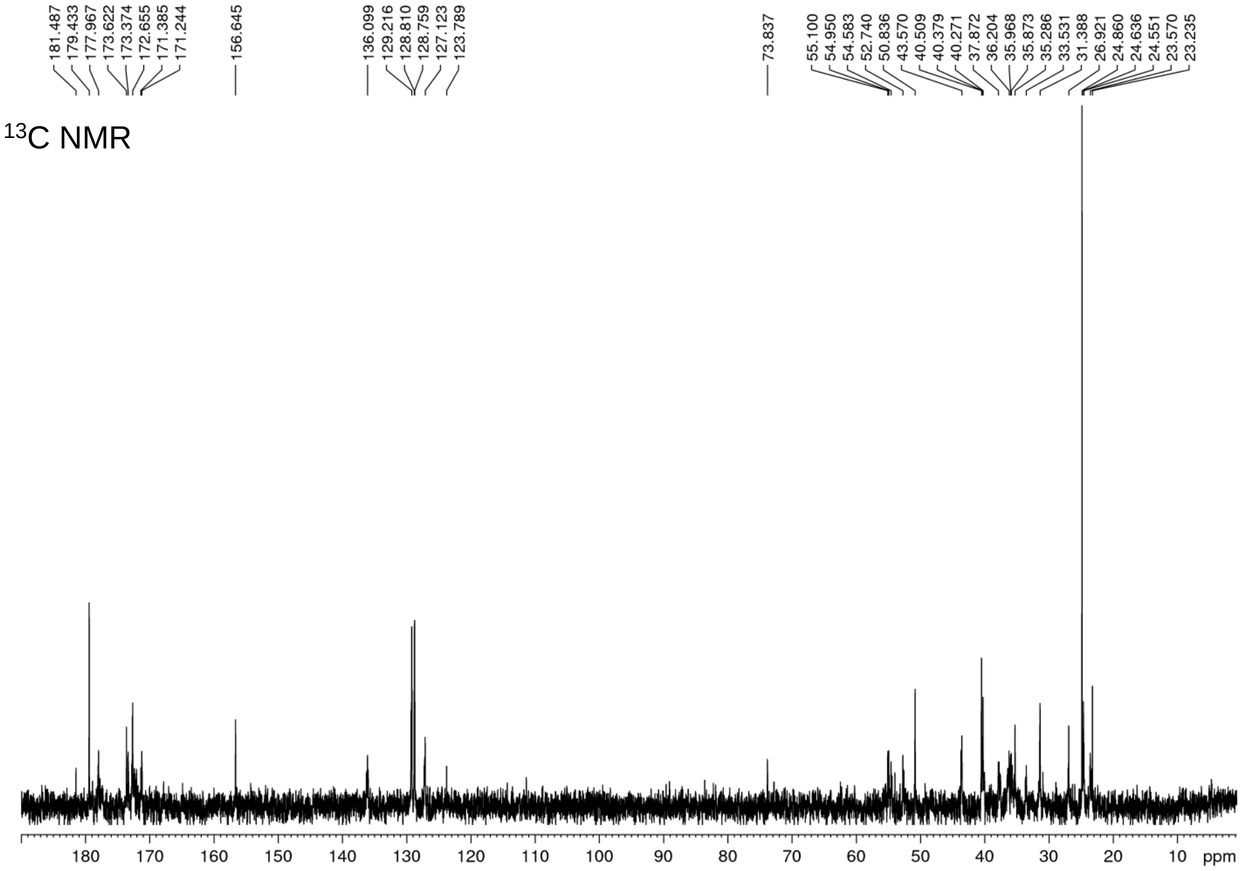
181.487
179.433
177.967
173.622
173.374
172.655
171.385
171.244
156.645
136.099
129.216
128.810
128.759
127.123
123.789
73.837
55.100
54.950
54.583
52.740
50.836
43.570
40.509
40.379
40.271
37.872
36.204
35.968
35.873
35.286
33.531
31.388
26.921
24.860
24.636
24.551
23.570
23.235
13C NMR
180 170 160 150 140 130 120 110 100 90 80 70 60 50 40 30 20 10 ppm

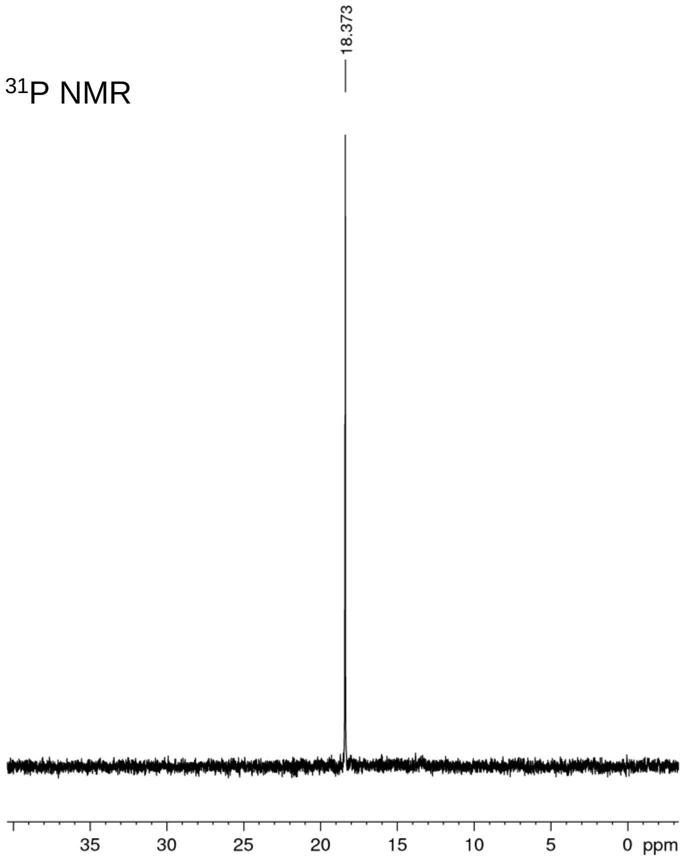
18.373
31P NMR
35 30 25 20 15 10 5 0 ppm

COSY HH

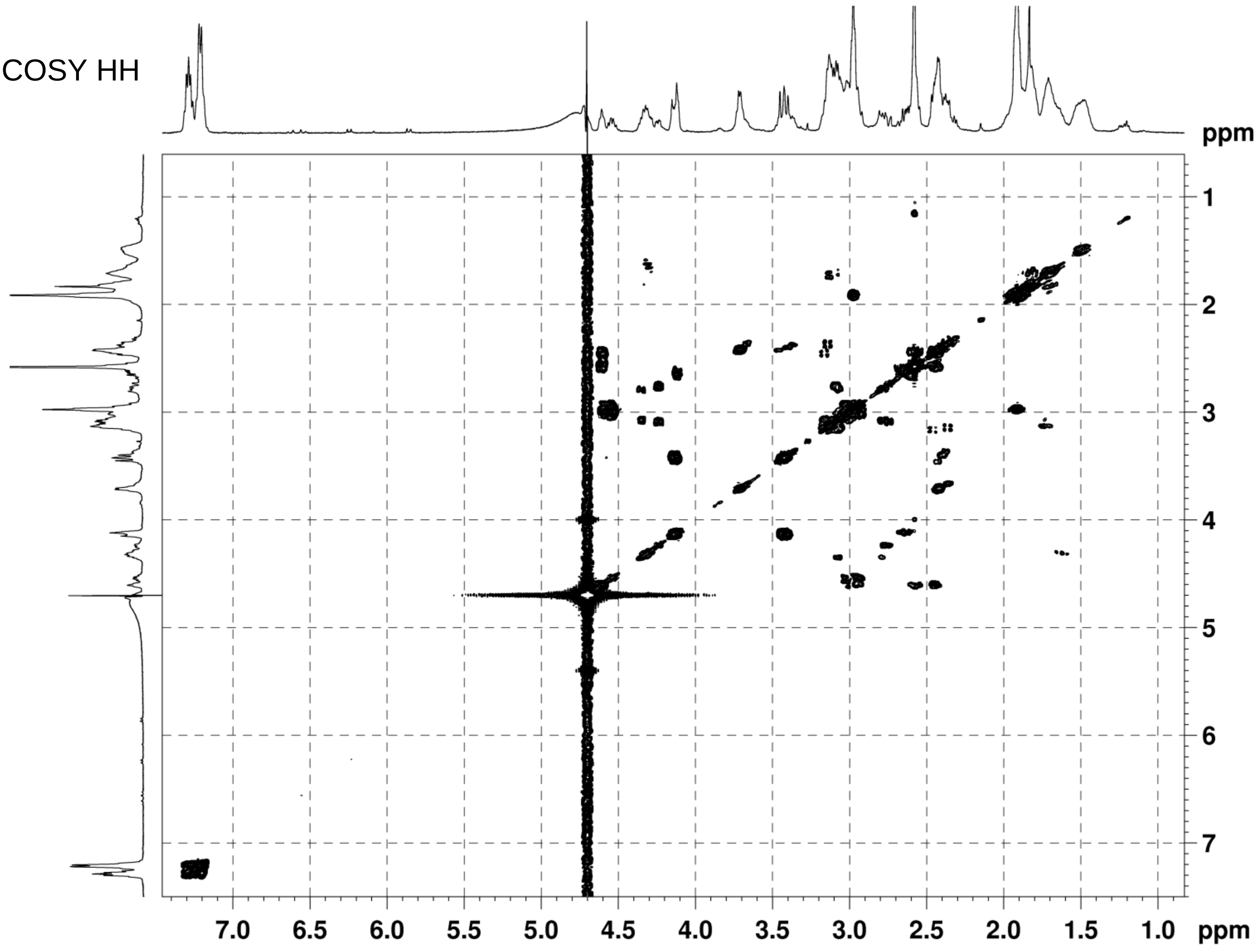

Figure S3. ^{1}H, ^{13}C, ^{31}P and COSY HH NMR of compound (**6b**).

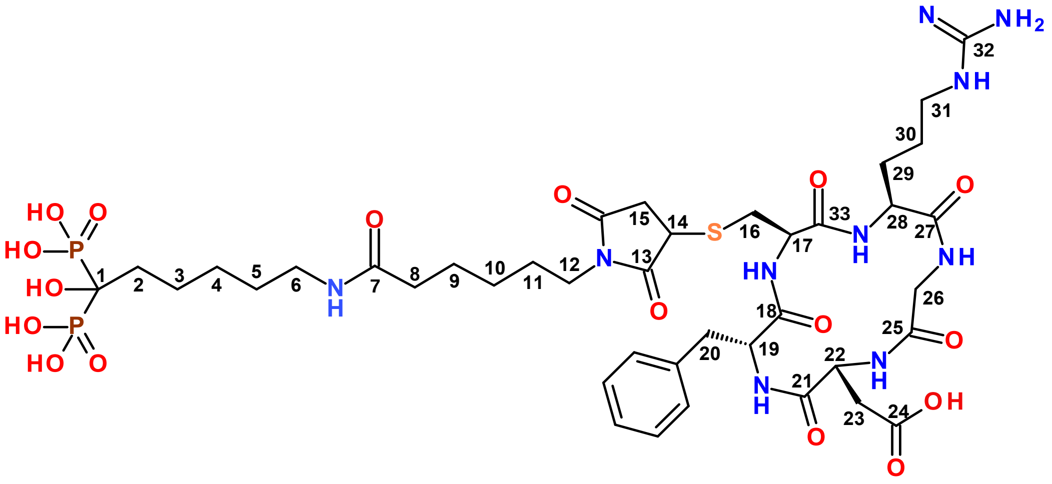

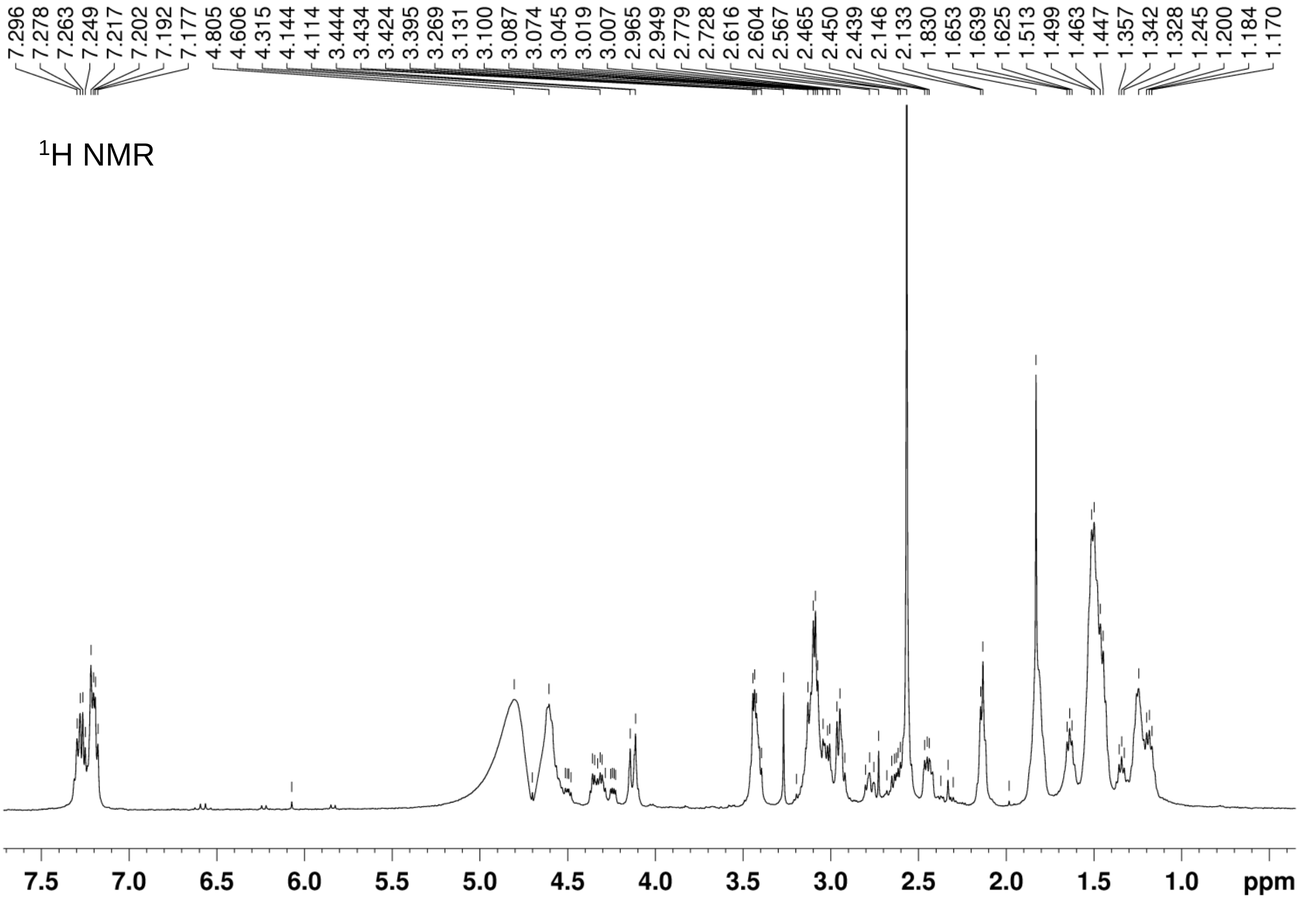

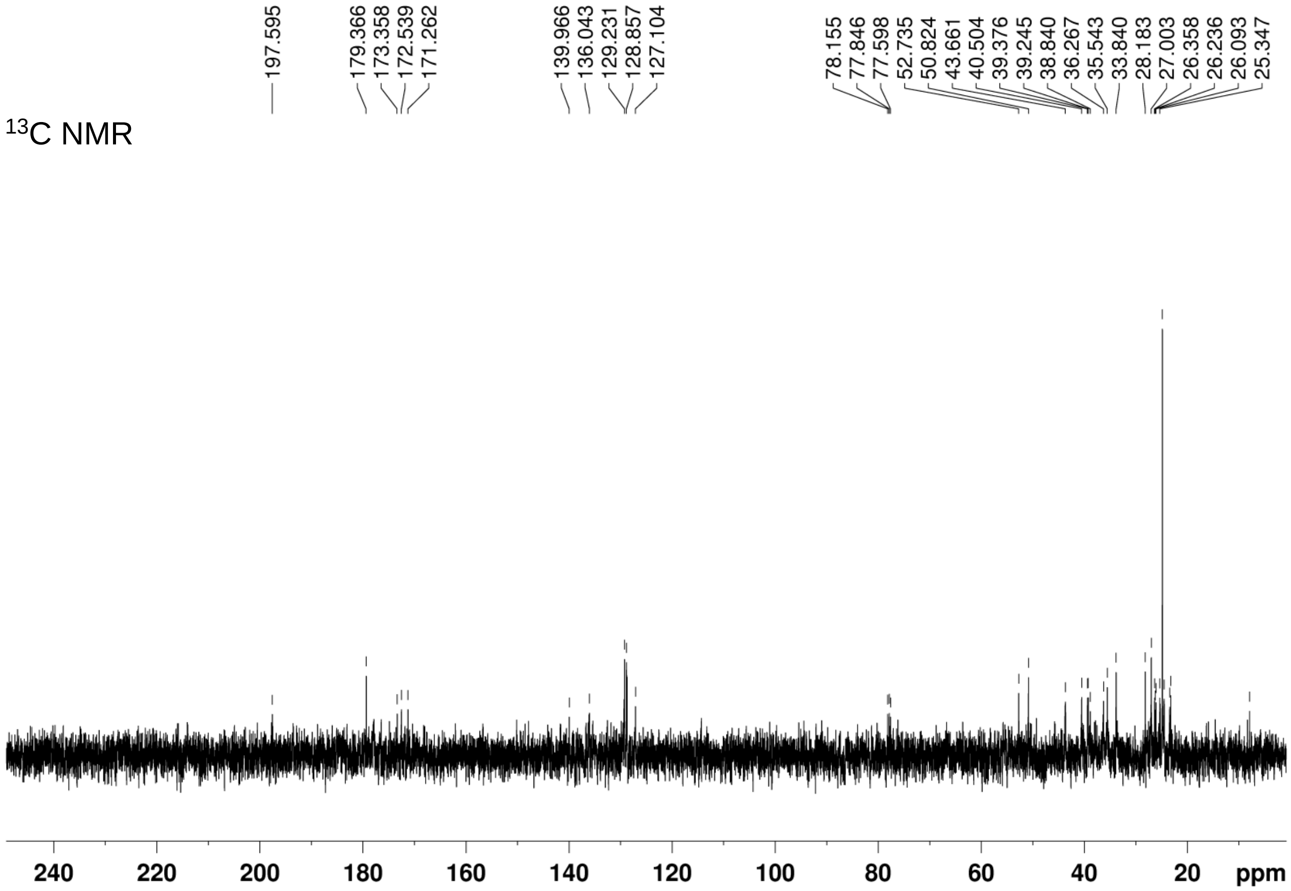
^{13}C NMR
197.595
179.366
173.358
172.539
171.262
139.966
136.043
129.231
128.857
127.104
78.155
77.846
77.598
52.735
50.824
43.661
40.504
39.376
39.245
38.840
36.267
35.543
33.840
28.183
27.003
26.358
26.236
26.093
25.347
240 220 200 180 160 140 120 100 80 60 40 20 ppm

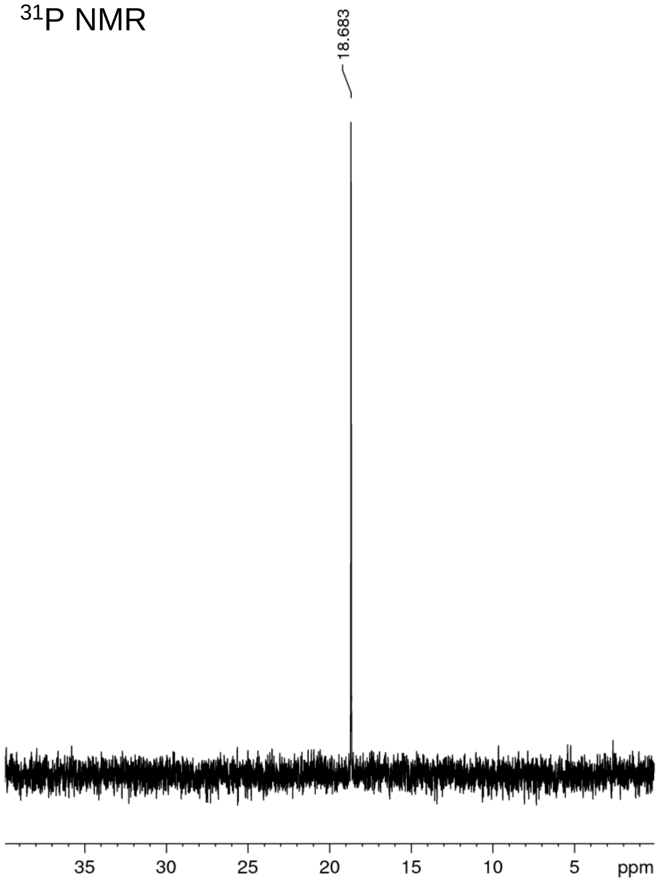
^{31}P NMR
18.683
35 30 25 20 15 10 5 ppm

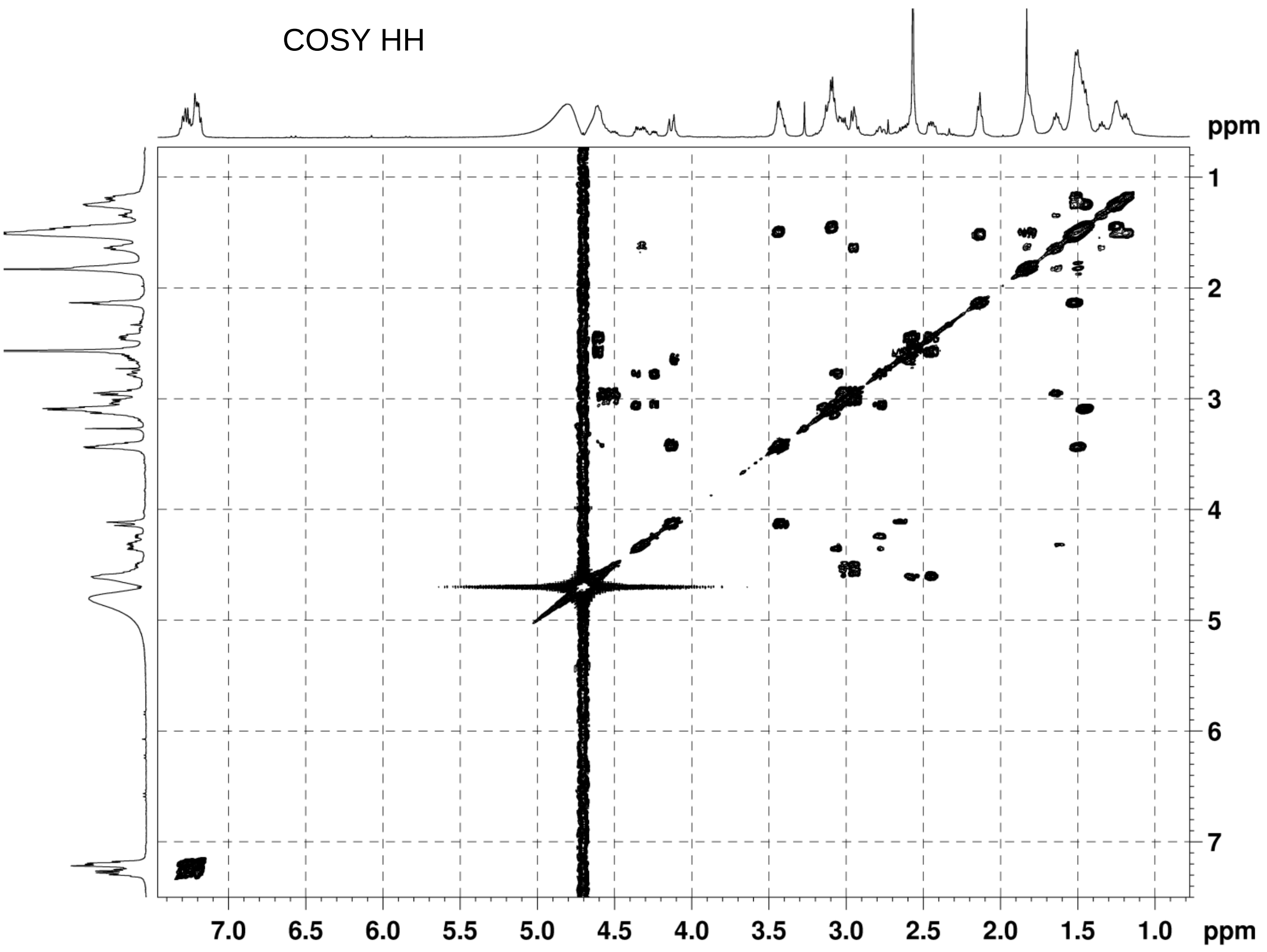

COSY HH
ppm
1
2
3
4
5
6
7
7.0
6.5
6.0
5.5
5.0
4.5
4.0
3.5
3.0
2.5
2.0
1.5
1.0
ppm

Figure S4. ^{1}H, ^{13}C, ^{31}P and COSY HH NMR of compound (**6c**).

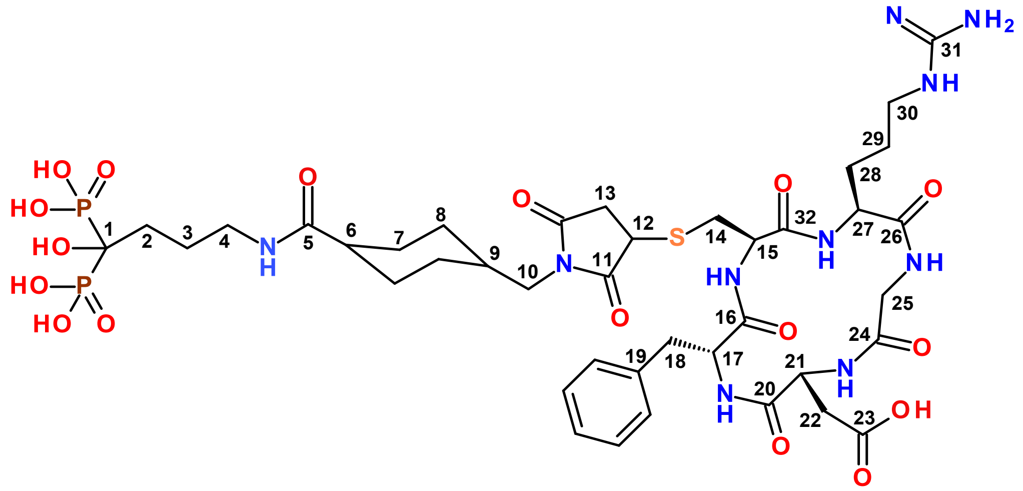

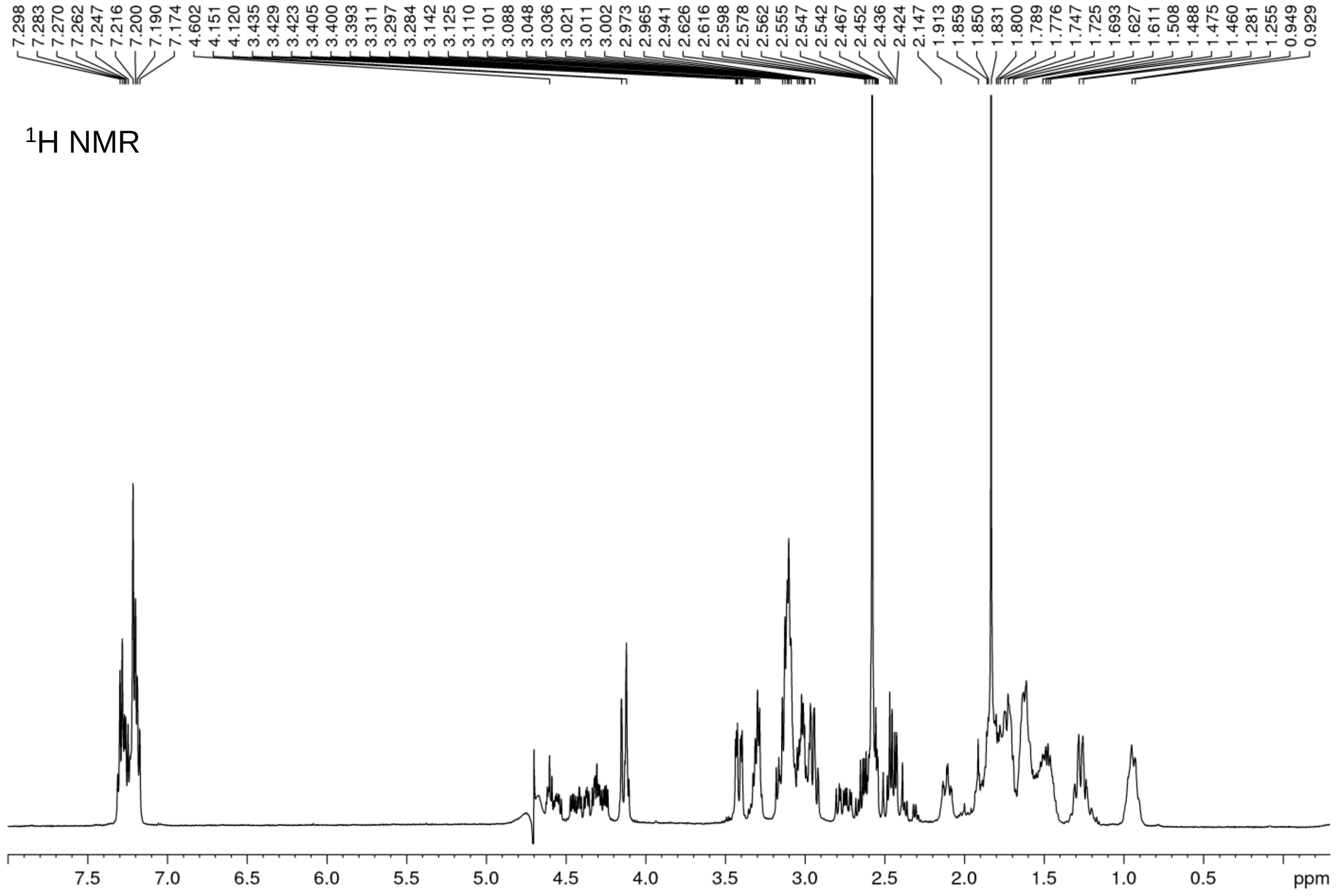

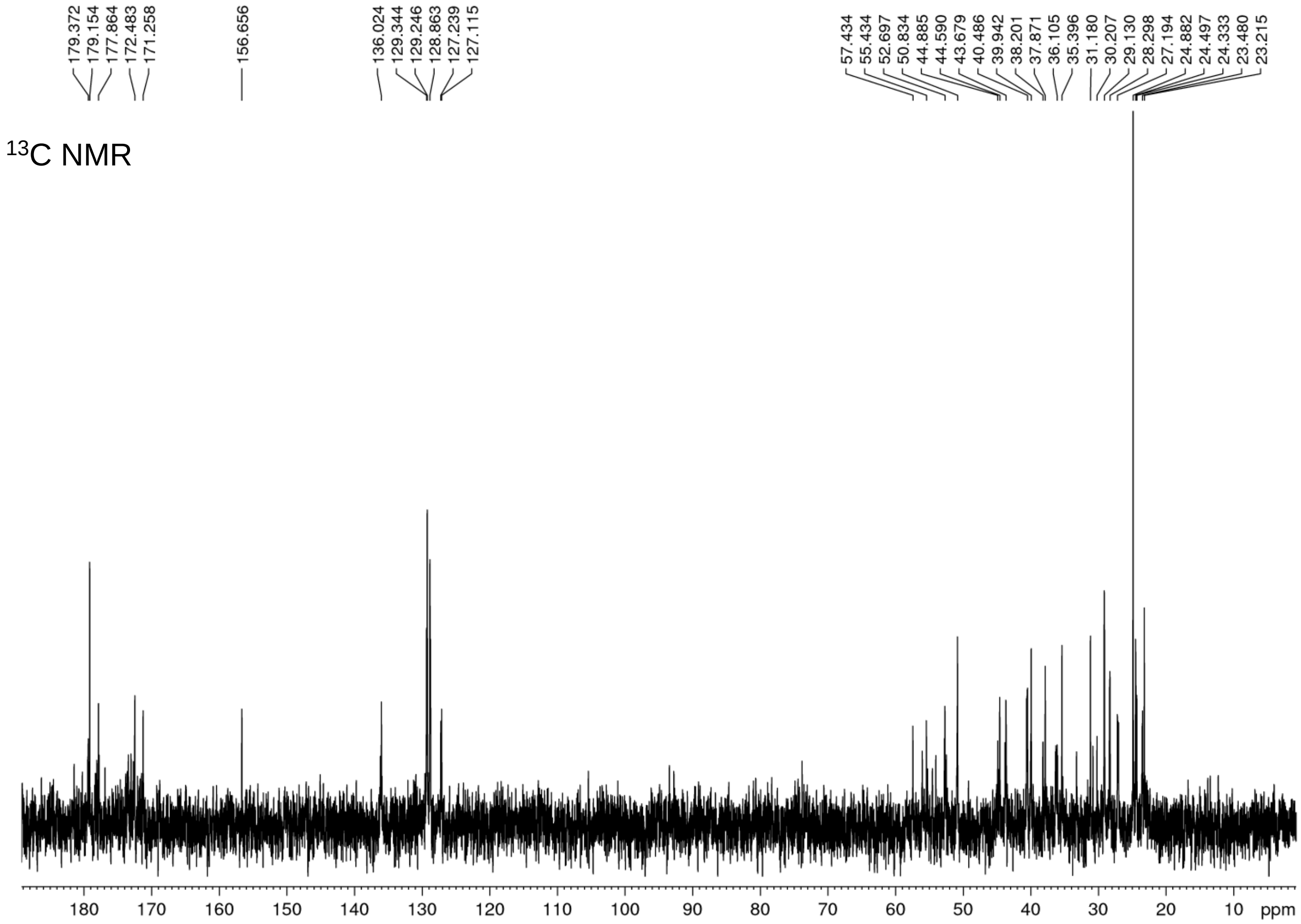

13C NMR
179.372
179.154
177.864
172.483
171.258
156.656
136.024
129.344
129.246
128.863
127.239
127.115
57.434
55.434
52.697
50.834
44.885
44.590
43.679
40.486
39.942
38.201
37.871
36.105
35.396
31.180
30.207
29.130
28.298
27.194
24.882
24.497
24.333
23.480
23.215
ppm

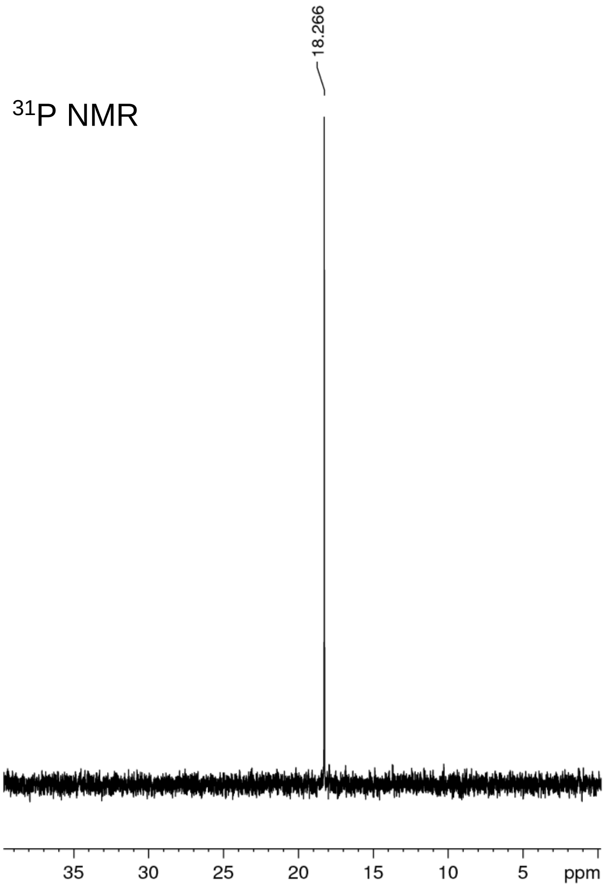

31P NMR
18.266
ppm

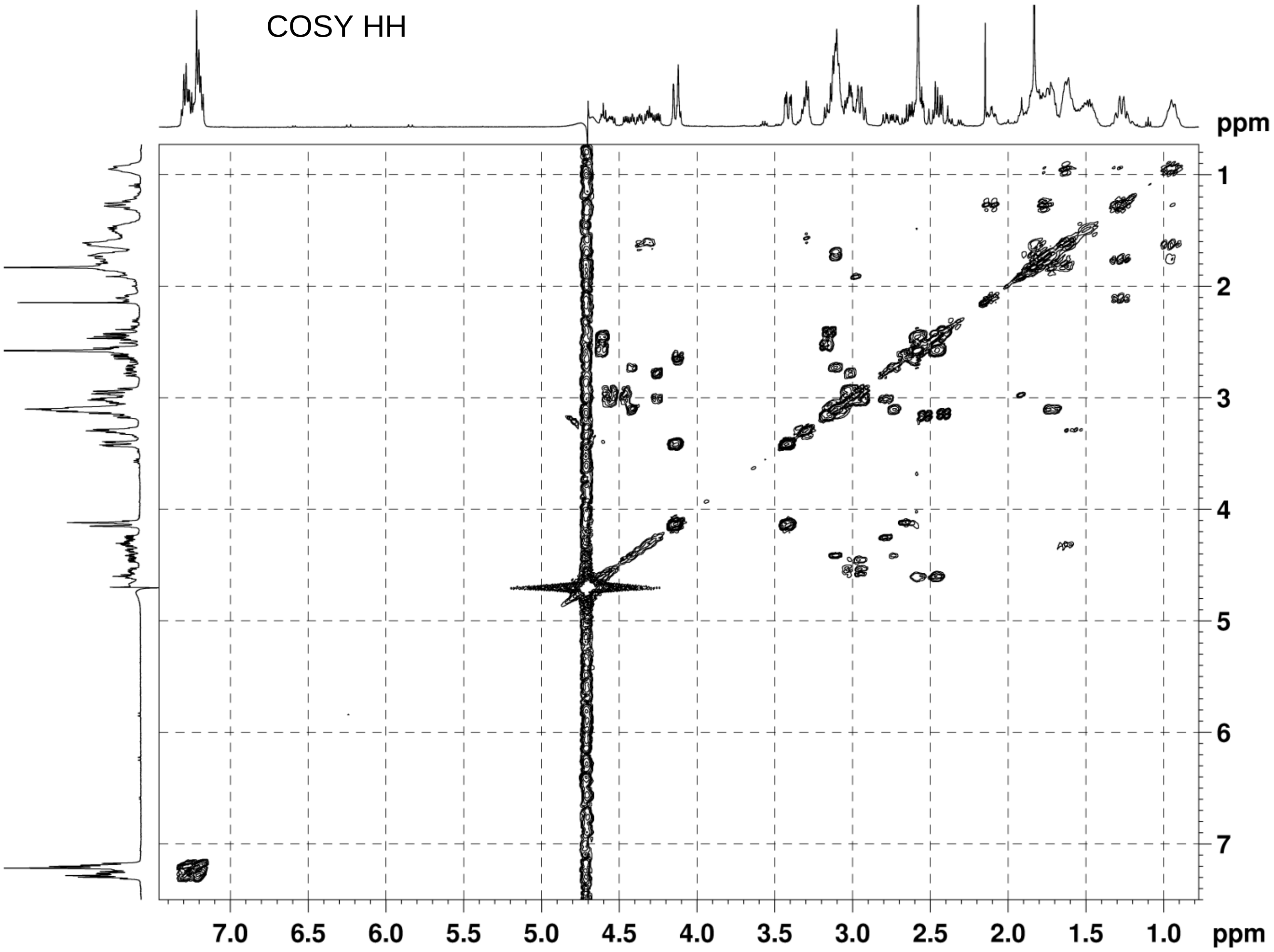
COSY HH
ppm
7.0
6.5
6.0
5.5
5.0
4.5
4.0
3.5
3.0
2.5
2.0
1.5
1.0
ppm
1
2
3
4
5
6
7

Figure S5. ^{1}H, ^{13}C, ^{31}P and COSY HH NMR of compound (**6d**).

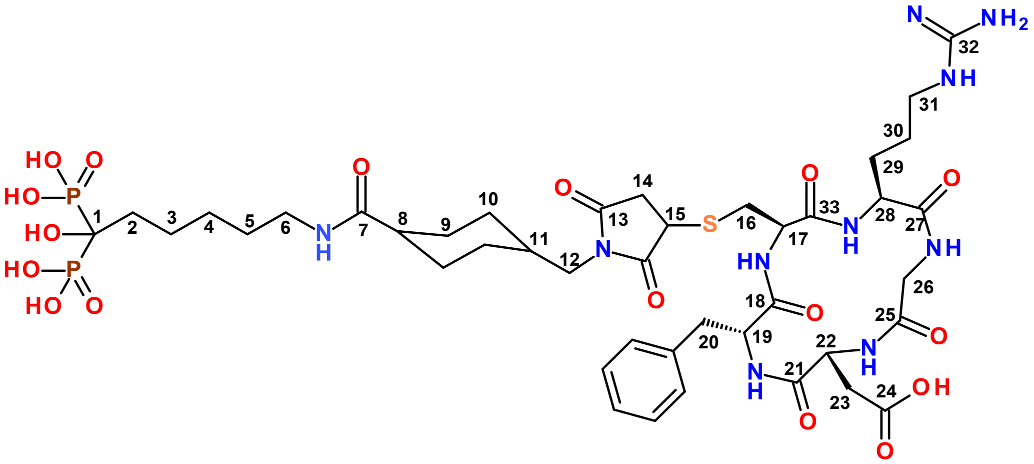

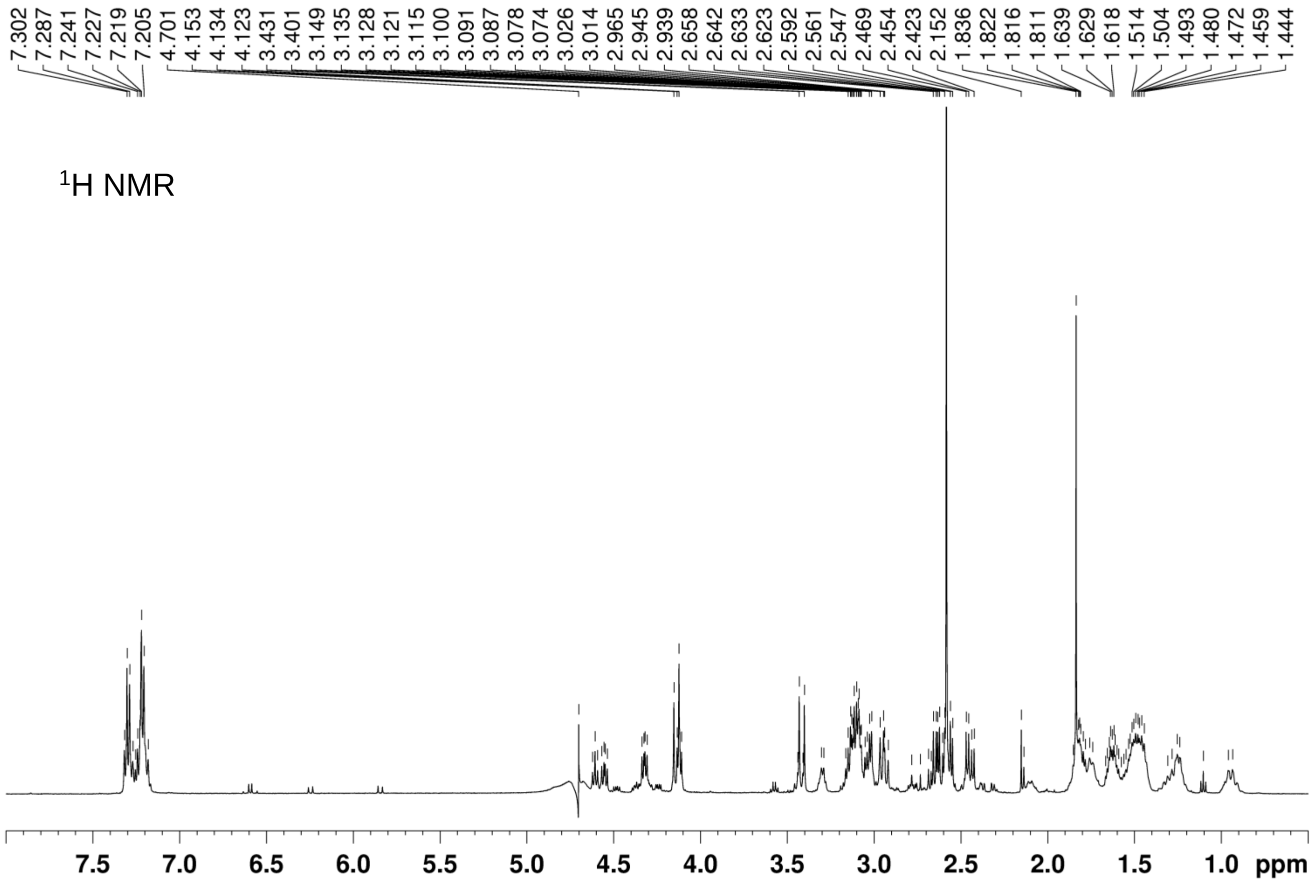

13C NMR

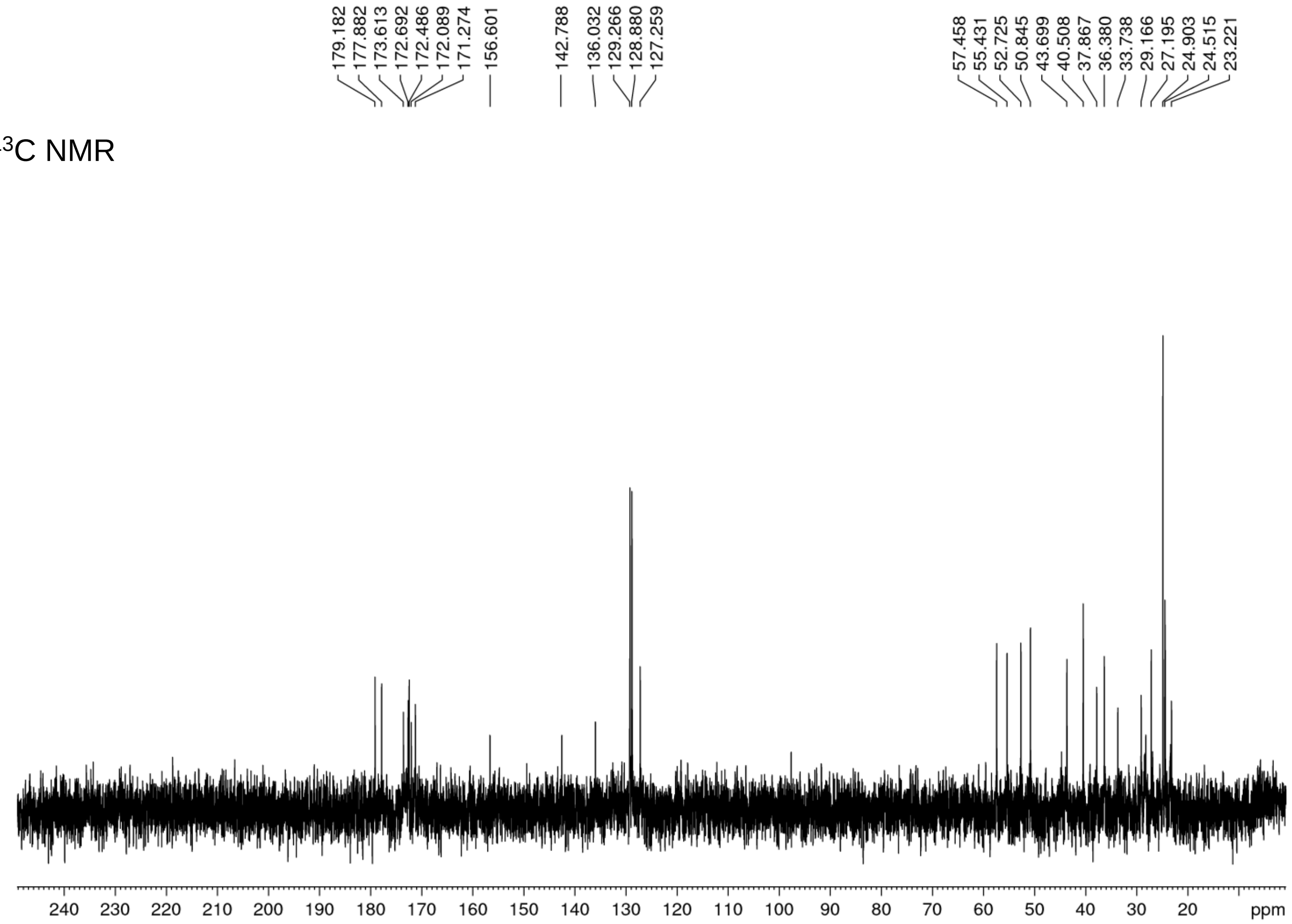
179.182
177.882
173.613
172.692
172.486
172.089
171.274
156.601
142.788
136.032
129.266
128.880
127.259
57.458
55.431
52.725
50.845
43.699
40.508
37.867
36.380
33.738
29.166
27.195
24.903
24.515
23.221
240 230 220 210 200 190 180 170 160 150 140 130 120 110 100 90 80 70 60 50 40 30 20 ppm

31P NMR

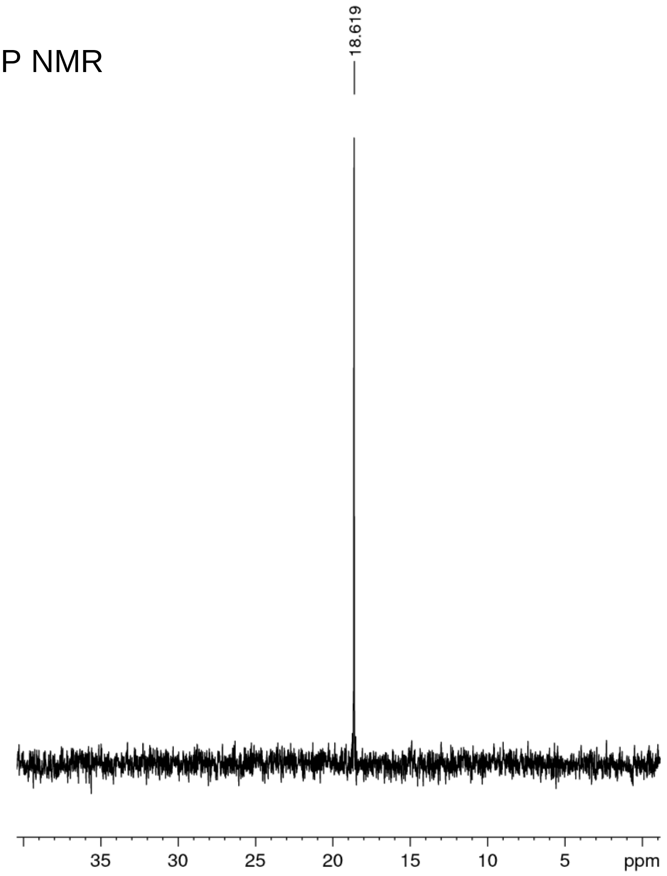
18.619
35 30 25 20 15 10 5 ppm

COSY HH

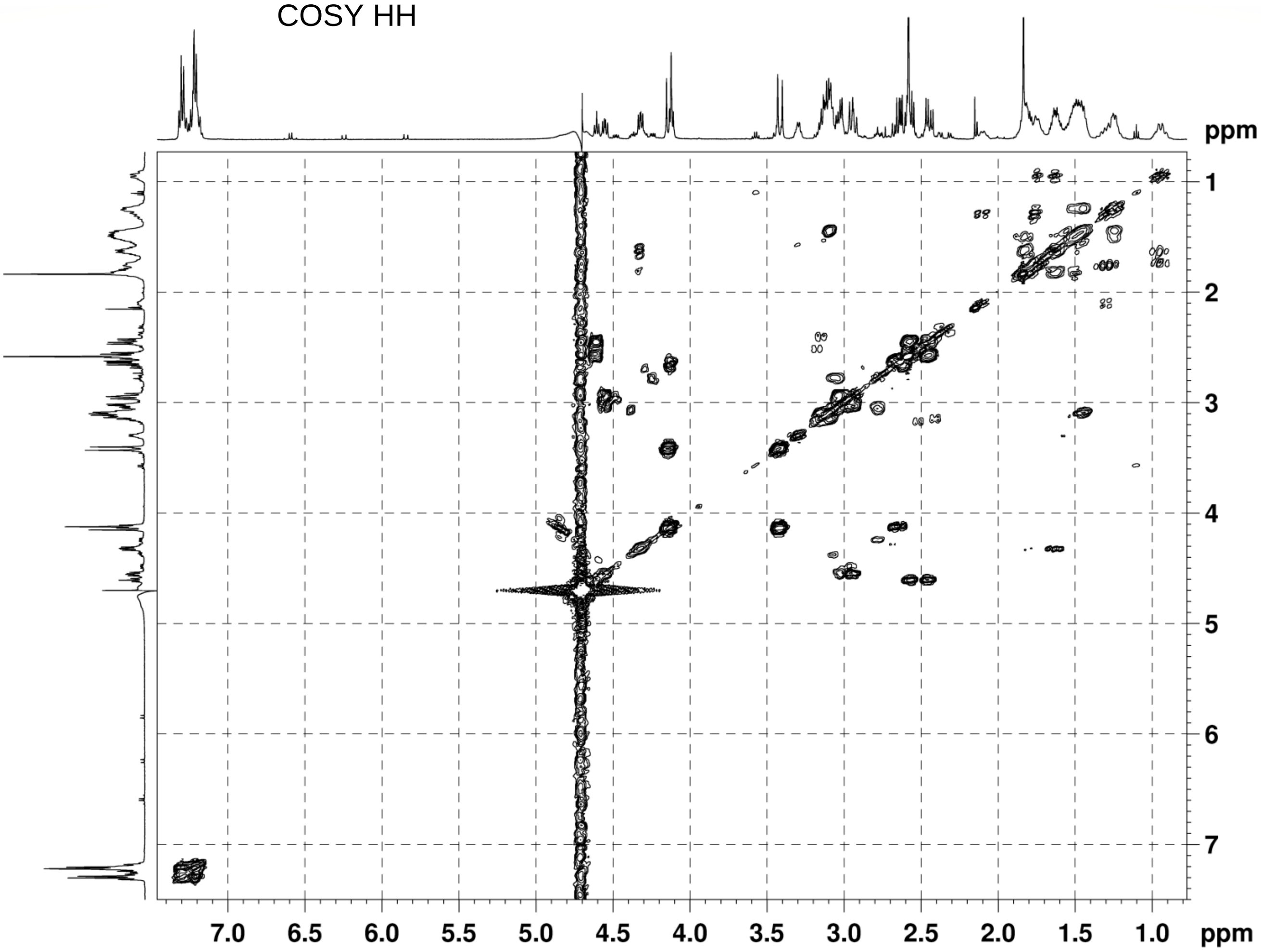

Supplement: Supplementary file 1 [file materials-15-08120-s001.zip › materials-1955109-supplementary.pdf]
